# Role of Sulfation of Zirconia Catalysts in Vapor Phase Ketonization of Acetic Acid

**DOI:** 10.1021/acs.jpcc.1c06920

**Published:** 2021-12-13

**Authors:** Maicon Delarmelina, Gunjan Deshmukh, Alexandre Goguet, C. Richard A. Catlow, Haresh Manyar

**Affiliations:** † School of Chemistry, Cardiff University, Main Building, Park Place, Cardiff CF10 3AT, United Kingdom; ‡ UK Catalysis Hub, Research Complex at Harwell, STFC Rutherford Appleton Laboratory, Didcot, Oxfordshire OX11 0FA, United Kingdom; § School of Chemistry and Chemical Engineering, Queen’s University Belfast, David-Keir Building, Stranmillis Road, Belfast BT9 5AG, United Kingdom; ∥ Department of Chemistry, University College London, 20 Gordon St., London WC1 HOAJ, United Kingdom

## Abstract

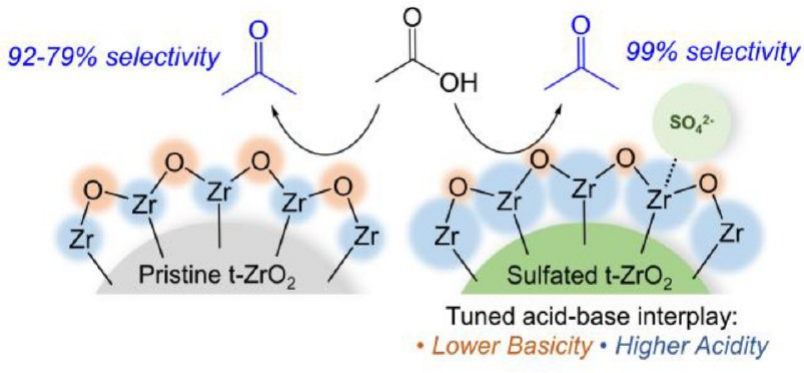

The effect of the sulfation of zirconia catalysts on their structure, acidity/basicity, and catalytic activity/selectivity toward the ketonization of organic acids is investigated by a combined experimental and computational method. Here, we show that, upon sulfation, zirconia catalysts exhibit a significant increase in their Brønsted and Lewis acid strength, whereas their Lewis basicity is significantly reduced. Such changes in the interplay between acid–base sites result in an improvement of the selectivity toward the ketonization process, although the measured conversion rates show a significant drop. We report a detailed DFT investigation of the putative surface species on sulfated zirconia, including the possible formation of dimeric pyrosulfate (S_2_O_7_^2–^) species. Our results show that the formation of such a dimeric system is an endothermic process, with energy barriers ranging between 60.0 and 70.0 kcal mol^–1^, and which is likely to occur only at high SO_4_^2–^ coverages (4 S/nm^2^), high temperatures, and dehydrating conditions. Conversely, the formation of monomeric species is expected at lower SO_4_^2–^ coverages, mild temperatures, and in the presence of water, which are the usual conditions experienced during the chemical upgrading of biofuels.

## 1 Introduction

Sulfated zirconia (SZ) is well established as a robust and efficient solid acid catalyst for a variety of reactions of high industrial importance, such as hydrocarbon isomerization, methanol conversion to hydrocarbons, alkylation, acylation, esterification, dehydration, and Fischer–Tropsch processes.^1−4^ More recently, sulfated zirconia has also emerged as a promising catalyst for several biorefinery processes, such as esterification, biodiesel preparation,^5−10^ furfural synthesis^11−16^ and conversion,^17−20^ condensation of ketones,^21^ glycerol utilization,^22−24^ and, conversion of CO_2_ to light olefins.^25^ Among the biorefinery processes, the upgrading of biomass derived volatile fatty acids (C_3_–C_6_) to corresponding ketones (ketonization reactions) is a promising strategy for the synthesis of biofuels.

Ketonization is a condensation reaction between two carboxylic acid molecules, involving the loss of one C atom as CO_2_ and one molecule of H_2_O. Ketonization leads to the formation of a new C–C bond and an increase in the energy density of the resulting oxygenates by decreasing their oxygen contents. The ketones produced can then be subsequently converted to C_5_–C_11_ hydrocarbons via aldol condensation, followed by their catalytic hydrogenation for use as biofuels. Both basic and acidic metal oxides such as ZrO_2_, TiO_2_, and CeO_2_ as well as mixed metal oxide catalysts have been studied to improve the conversion of carboxylic acids and their selectivity to ketone.^26−29^ In this context, the use of amphoteric catalysts can be the key in tuning the strength of acid–base sites available for ketonization. Pristine zirconia (monoclinic and tetragonal ZrO_2_) has been investigated for the ketonization of acetic acid.^30,31^ However, even for this simple system, the mechanistic aspects of the ketonization reaction are not well understood, with both Eley–Rideal and Langmuir–Hinshelwood models being proposed and the role of the catalyst structure, strength and type of the acid–base sites, and its interaction with the reactant species poorly established. Sulfated zirconia has been more widely utilized as catalyst than pristine zirconia due to its enhanced acidity.^32^ However, the role of the sulfation of zirconia catalysts and the effect of the coverage, strength, and number of acid–base sites and the correlation of structure with activity and selectivity in the ketonization of acids has not been explored in detail. Establishing the structure–activity correlation by identifying the type and coverage of sulfated species, such as monomeric versus dimeric pyrosulfate species on zirconia will provide the basis of rational design criteria for tuning the catalyst activity and selectivity in ketonization reactions.^33^

The identity of S-containing species on sulfated zirconia and their impact over the amphoteric properties have been widely investigated by different authors and they are still a matter of debate. It has been widely accepted that the characterization of the surface species in these systems has not been unambiguously determined, and they are highly dependent on the experimental conditions employed and lack understanding at the molecular level.^1^ Li et al.^34,35^ have proposed that “labile” SO_3_/pyrosulfate-type surface species are responsible for the activity of sulfated zirconia in the isomerization of alkanes. The authors observed that water washing of the catalyst led to the removal of 40% of the sulfate content in the catalyst (the so-called labile portion of sulfates) with subsequent loss of activity. Additional XANES and thermogravimetric analysis led to similar conclusions.^36−39^ Vibrational spectroscopy of sulfated zirconia (SZ) has also been widely used for the characterization of the material, often together with computational approaches; literature data are summarized in Table 1.

**Table 1:** Selected Examples of Previously Reported Vibrational Spectroscopy Investigation of Sulfated Zirconia Systems by Experimental and Computational Approaches

system	coverage (SO_x_ nm^-2^)	method	wavenumber (cm^-1^)	ref
t-ZrO_2_(101)	2	DFT-PBE^a^	possible species on the surface:	(40)
			[2H^+^, SO_4_^2–^]: 1375 (ν_S=O_);1010, 978, 924 (ν_S–O_)	
			[H^+^, HSO_4_^–^]: 1412 (ν_S=O_);1221, 1056, 698 (ν_S–O_)	
t-ZrO_2_(001)	2		possible species on the surface:	
			[H^+^, OH^–^, SO_3_]: 1425 (ν_S=O_); 1056, 1000, 943 (ν_S–O_)	
			[2H^+^, SO_4_^2–^]: 1429 (ν_S=O,Asym_), 1344 (ν_S=O,Sym_); 1235, 967 (ν_S–O_)	
noncalcinated t-SZ (Y-stabilized)^b^	5	FTIR^d^	3765 (w, ν_OH,Terminal_), 3675 (ν_OH,Brigded_), 3600 (shoulder), 1385, 1025	(41)
calcinated t-SZ (Y-stabilized)^c^	3	FTIR^d^	3760 (w, ν_OH,Terminal_), 3665 (ν_OH,Brigded_), 1390, 1010	
noncalcinated t-SZ (Y-stabilized)^b^^,^^e^	5	FTIR	1350–1390 (ν_S=O_), 1025 (ν_S–O_)	(42)
calcinated t-SZ (Y-stabilized)	3	FTIR	1350–1390 (ν_S=O_), 1025 (ν_S–O_)	(42)
t-ZrO_2_(101)	2	DFT-PW91^f^	possible species on the surface:	(43)
			[SO_3_]: 1398 (ν_Zr(s)OS=O_); 1029 (ν_Zr(w)OS-O_); 1004 (ν_Zr(s)OS-O_); 1001 (ν_Zr(s)OS-O_)	
			[SO_3_, OH^–^, H^+^, H_2_O]: 1324 (ν_Zr(s)OS=O_); 1118, 1011 (ν_Zr(s)OS-O_); 935 (ν_Zr(w)OS-O_)	
			[SO_4_^2–^, 2H^+^, 3H_2_O]: 1310 (ν_Zr(s)OS=O_); 1143, 1111 (ν_S–O_ + δ_Zr–O–H_); 935 (ν_Zr(s)OS-O_)	
t-ZrO_2_(101)	4	DFT-PW91^f^	possible species on the surface:	(43)
			[HSO_4_^–^, SO_4_^2–^, 3H^+^, 2H_2_O]: 1474, 1374 (ν_Zr(w)OS=O_); 1272, 1262, 1195 (ν_Zr(s)OS=O_); 998 (ν_Zr(w)OS-O_); 951 (ν_Zr(s)OS-O_)	
			[S_2_O_7_^2–^, 2H^+^, H_2_O]: 1421 (ν_Zr(s)OS=O_); 1317 (ν_Zr(w)OS=O_); 1214 (ν_Zr(s)OS=O_); 1174, 1112 (ν_Zr(w)OS-O_ + δ_Zr–O–H_); 1084, 999 (ν_Zr(s)OS-O_ + ν_Zr(w)OS-O_); 901, 889 (ν_S–O_ + δ_Zr–O–H_)	
			[S_2_O_7_^2–^, 2H^+^]: 1417 (ν_Zr(s)OS=O_);1401 (ν_Zr(w)OS=O_ + ν_Zr(w)OS-O_); 1131, 1045 (ν_Zr(w)OS-O_);1199, 992, 969 (ν_Zr(s)OS-O_)	

a Calculated frequencies were scaled by 1.05.

b Thermal treatment in air at 400 °C after sulfation.

c Thermal treatment in air at 650 °C after sulfation.

d Vacuum activated at 400 °C.

e Inactive toward isomerization of *n*-butane.

f Calculated frequencies were scaled by 1.07376.

Early DFT investigations by Haase and Sauer^40^ showed that the adsorption of sulfate groups over t-ZrO_2_ (101) and (001) surfaces occurs preferentially in a 3-fold or 2-fold fashion (tridentate or bidentate sulfate groups), respectively. Later, Hofmann and Sauer^43^ studied the adsorption for different combinations and loadings of H_2_O and SO_3_ or H_2_SO_4_ over t-ZrO_2_ (101). First, the adsorption of one H_2_O molecule was considered up to a full monolayer over a 2 × 1 t-ZrO_2_ (101) model surface (four surface Zr, two strong and two weak Lewis acids, as discussed later in section 3.2). Next, SO_3_ adsorption (up to two molecules) was investigated, in which, the formation of an additional bond between the sulfur atom and a surface oxygen atom was observed upon relaxation. Finally, the authors modeled coadsorption of SO_3_ and H_2_O, considering separate SO_3_ and H_2_O species and dissociated H_2_SO_4_. The calculated adsorption energies show that the [SO_3_,H^+^,OH^–^] configuration (*E*_ads_ = −370 kJ/mol) is favored over [H^+^,HSO_4_^–^] and [2H^+^,SO_4_^2–^] (*E*_ads_ = −292 and −315 kJ/mol, respectively). By thoroughly considering adsorption of two H_2_SO_4_ species and adsorption of the H_2_S_2_O_7_ dimer at different pressures and temperatures on a 1 × 2 t-ZrO_2_ (101) surface (roughly, a coverage of 4 S/nm^2^), these authors suggested that water-rich structures, [SO_4_^2–^,2H^+^,3H_2_O] over t-ZrO_2_ (101) are likely to be converted into pyrosulfate structures, [S_2_O_7_^2-,^2H^+^,H_2_O], during calcination. Kanougi et al.^44^ also investigated the structure of SZ and observed a new SO_3_ adsorption structure, formed through the abstraction of one surface oxygen by SO_3_ to produce a sulfate-like species on the surface. Hofmann and Sauer suggested, however, that such a possibility may not occur in a water-rich environment, as under these conditions the formation of SO_3_ is unlikely to occur.^43^ It is worth mentioning that, as highlighted by Sauer et al., the characteristic vibrational band assigned to pyrosulfate above 1400 cm^–1^ is only observed at high S coverages (4 S/nm^2^);^45^ for lower coverages (1–2 S/nm^2^) the composition of the surface species may differ.

In this study, we have combined experimental data from catalytic activity studies in the acetic acid ketonization with theoretical calculations to correlate the coverage and type of sulfated species present on the zirconia surface with activity and selectivity. We investigate the effect of different H_2_SO_4_ loadings on the structure and catalytic activity of these materials, as well as explore by DFT methods the most stable configurations of the S-containing species on the surface of the catalysts. Overall, these results provide an understanding of the structure of sulfated zirconia-based systems, putative active species over their surface, and their acid–base properties. Moreover, our results offer key insights into catalyst design criteria which can be further extended to other biorefinery processes, as well as for the development of alternative approaches to mitigate possible deactivation mechanisms and for the preparation of new sulfated zirconia-based systems with maximized efficiency.

## 2 Methodology

### 2.1 Experimental Methods

#### 2.1.1 Catalyst Preparation

All catalysts were prepared by a precipitation method. A desired amount of ZrO(NO_3_)_2_.H_2_O was placed in a glass vessel and dissolved in distilled water. The solution was stirred using a magnetic stirrer to obtain a clear solution before a 25% aqueous NH_3_ solution was added dropwise during 30 min to obtain a white precipitate while the pH of the solution was maintained at 8.5. The precipitate was stirred for 1 h before the precipitate was left to settle. The precipitate was subsequently washed with distilled water by using the settle and decant method until a neutral pH for the supernatant liquid was obtained. The resultant solid was filtered, dried at 120 °C, and finally calcined at 500 °C. Sulfated catalysts were prepared by stirring the as synthesized Zr(OH)_4_ in 0.1, 0.5, and 1 mol L^–1^ H_2_SO_4_ solution (15 mL g^–1^) for 12–15 h, followed by filtration and washing with distilled water, drying at 120 °C, followed by calcination at 500 °C. All catalyst samples were sieved to obtain a fine powder, with particle sizes smaller than 45 μm before being subjected to the activity tests in order to avoid any mass transfer limitations.

#### 2.1.2 Catalyst Activity

**Figure 1:**
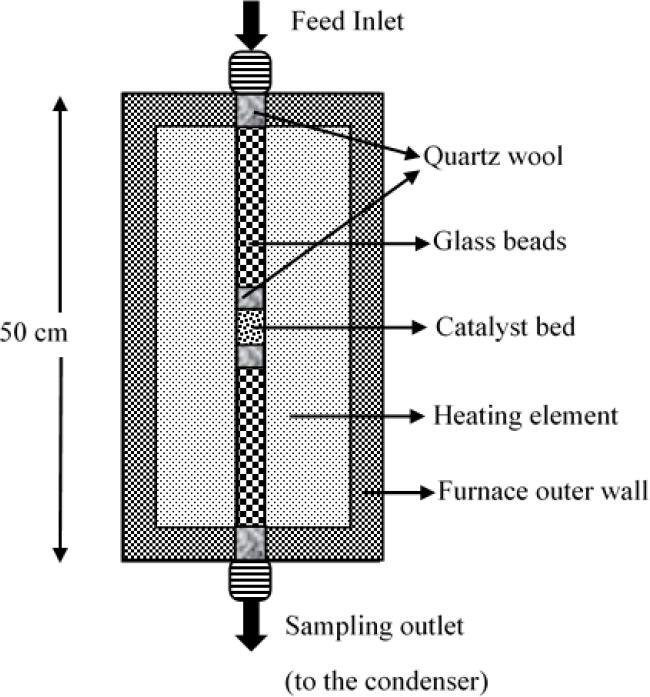
Cross section of fixed bed reactor used for ketonization of acetic acid.

The catalytic activity of the sulfated zirconia catalysts was evaluated in the ketonization of acetic acid, as a model substrate. The reaction was performed in a fixed bed reactor (50 cm length and 0.9 cm inner diameter). The catalyst was packed in the middle of the reactor between quartz wool plugs (Figure 1) with glass beads used as inert packing material. The reactor was first heated to 350 °C for 30 min under nitrogen flow before the gas flow was stopped and acetic acid was fed into the system using a HPLC pump (Shimadzu LC 20AD). The blank reaction without catalyst was studied with reactor inert material (glass beads and quartz wool). No conversion of acetic acid was observed at 350 °C with 0.1 mL min^–1^. Samples were collected at the outlet of the reactor using a gas–liquid separator and were analyzed by gas chromatography fitted with an Innowax capillary column (60 m) and flame ionization detector.

To confirm the absence of mass transport resistance, we have calculated both the external resistance coefficient (*f*_ex_) and Wiesz–Prater criterion (*C*_wp_) to assess the influence of external mass transfer resistance as well as intraparticle diffusion resistance.^46−48^ In all cases for the three 0.1, 0.5, and 1 SZ catalysts, the value of external resistance coefficient (*f*_ex_) was in the range of 1.1 × 10^–5^ to 1.4 × 10^–5^, which was much less than 1, confirming there was no external mass transfer resistance.^46,47^ According to the Wiesz–Prater criterion, the dimensionless parameter (*C*_wp_) which represents the ratio of the intrinsic reaction rate to the intraparticle diffusion rate, in all cases for the three 0.1, 0.5, and 1 SZ catalysts, the value of *C*_wp_ was in the range of 1.5 × 10^–3^ to 3.3 × 10^–3^, which was much less than 1, and therefore, it proved that there was no intraparticle diffusion resistance.^48^

#### 2.1.3 Catalyst Characterization

XRD analyses were performed on a Panalytical X-Pert Pro MPD diffractometer with Ni filtered CuKα radiation (1.5405 A°). The diffractograms were recorded with a step size of 0.016° from 5° to 80°. FT-IR spectra were measured using an Agilent Cary 630 FT-IR spectrometer with 32 scans per sample. The surface area and pore volume were determined from the N_2_ adsorption–desorption isotherms at 77 K using a Micromeritics ASAP 2010. The samples were degassed at 393 K, under vacuum, for 24 h carried out before nitrogen adsorption studies. The acidic sites on the surface of the pristine and sulfated catalyst were calculated by titration method. All of the catalysts were stirred in 0.1 M NaOH solution for 6 h to neutralize the surface acidity of the catalyst, and unreacted NaOH was titrated against 0.1 M HCl solution to get an account of surface acidity. The CO_2_ temperature programmed desorption (TPD) analysis of the catalyst was performed using the Micromeritics AutoChem II instrument.

### 2.2 Computational Methods

All calculations were performed using the Vienna ab initio simulation package (VASP) within the framework of periodic density functional theory (DFT). The electronic structure of all systems modeled employed the RPBE functional combined with Grimme’s semiclassical D3 dispersion correction and Coulomb repulsive interaction (*U* = 4 eV) for *d* orbitals of Zr, in accordance with our previous publication.^49^ The electron–ionic core interaction was represented by the projector-augmented-wave (PAW) potentials and the cutoff energy was selected after extensive benchmarking and set to 550 eV.^49^ The Zr 4s^2^4p^6^4d^2^5s^2^ and O 2s^2^2p^4^ orbitals were explicitly included as valence electrons. Brillouin zone sampling was performed using the Monkhorst–Pack scheme with a k-point grid of 5 × 5 × 1 together with a Gaussian smearing broadening of 0.02 eV. Forces and electronic SCF convergence were set at 10^–2^ eV Å^–1^ and 10^–5^ eV, respectively.

The slab model for the t-ZrO_2_ (101) surface used a 2 × 2 × 3 supercell containing 24 zirconium and 48 oxygen atoms, which was constructed from the optimized bulk structure, in accordance with our previous publication.^49^ The resulting structure presented 3 O–Zr–O trilayers, of which the top two were allowed to relax in all optimizations. A vacuum box of 15 Å in the *z* direction was added to the surface in order to avoid undesired interactions with slab images. Further details are given in the Supporting Information (Figure S1).

Transition state structures and calculated energy barriers were computed by preliminary optimization using the climbing image nudged elastic band method (CI-NEB) method,^50^ followed by final optimization using the Improved Dimer method (IDM).^51,52^ All investigated reaction coordinates were sampled by eight images and sampling of the reciprocal space was limited to the Γ-point. Next, the obtained transition state structures were confirmed to be a saddle-point in the potential energy surface by vibrational frequency calculation and observance of a single imaginary vibrational mode corresponding to the investigated reaction coordinate.

All reported adsorption energies (*E*_ads_) were calculated using eq 1, where *E*_(Clean Surface)_ is the total energy of the clean surface, *E*_(Adsorbate)_ is the energy of the adsorbate in the 15 Å × 15 Å × 15 Å vacuum box, and *E*_(Surface+Adsorbate)_ is the energy of the surface interacting with the adsorbate. Vibrational frequencies were calculated for selected CO_2_^-^ and pyridine-containing surfaces and these results are available in the Supporting Information (Tables S3–S6).

The Brønsted acidity of the surfaces was estimated by calculation of the required energy to transfer a proton from the protonated surface site to a water molecule put in the vacuum between slabs (*E*_H+,transf_.), according to eq 2. The term *E*(O_Surf_-H_;_ H_2_O) is the energy of the protonated surface with one neutral water molecule in the vacuum between slabs; the term *E*(O_Surf_^–^; H_3_O^+^) is the energy of the system after proton transfer from the surface to the water molecule.
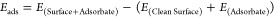
1
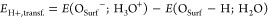
2

## 3 Results and Discussion

### 3.1 Synthesis of the Catalysts: The Effect of Distinct SO_4_^–2^ Concentrations

**Figure 2:**
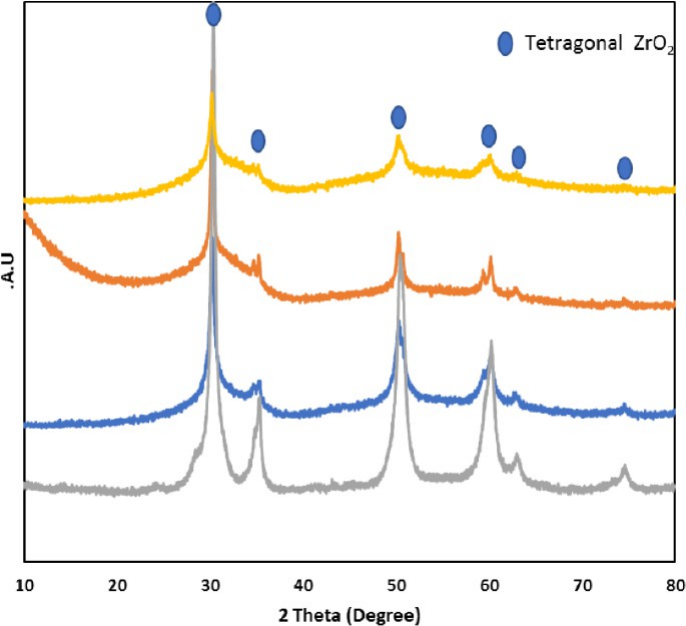
XRD diffraction patterns of pristine and sulfated zirconia catalysts calcined at 500 °C. Gray line: ZrO_2_, Blue: 0.1 SZ, Red: 0.5 SZ, and Gold: 1 SZ.

The XRD patterns of all of the catalysts prepared are reported in Figure 2. The synthesized sulfated zirconia (0.1, 0.5, and 1 SZ) when calcined at 500 °C, resulted in the tetragonal phase with diffraction peaks with 2θ values at 30.3, 35.2, 50.4, 60.2, 63, and 74.2 which are attributed to the tetragonal phase of ZrO_2_ (JCPDS-17-0923; Figure 2).^53,54^ The XRD pattern of the synthesized ZrO_2_ catalyst was compared with sulfated catalysts. Sulfation of the catalyst does not affect the phase of the catalyst but reduces the crystallinity and the sharpness of the peaks which is probably due to the coverage of S species on the surface of the catalyst affecting crystallite size.

**Figure 3:**
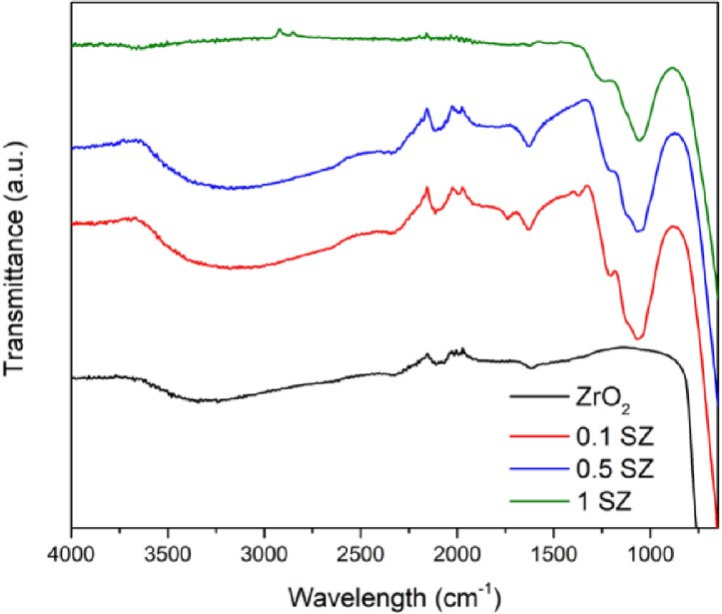
FT-IR spectra of pristine and sulfated zirconia catalysts.

The FT-IR spectra measured for both pristine and sulfated zirconia catalysts are shown in Figure 3. The broad peak at 3500–3000 cm^–1^ was assigned to −OH groups, and the peak at 1636 cm^–1^ was attributed to the bending mode of H_2_O molecules associated with sulfate groups.^20^ The peaks in the range 1300–900 cm^–1^ are attributed to the sulfate groups present on the catalyst surface. Only the sulfated catalysts showed characteristic peaks at 1323, 1289, 1111, 1051, 1015, and 975 cm^–1^ commonly associated with the vibrational frequencies of bidentate or tridentate SO_4_^2–^ species coordinated to surface Zr cations as previously reported.^55,56^

The detailed structural characterization of catalysts prepared including the BET surface area, pore size and pore volume measurement, crystallite size calculated using Scherrer equation and total surface acidity measured are shown in Table 2. The sulfation of the zirconia catalysts resulted in the linear increase in the surface area, along with the decrease in observed crystallite size.

**Table 2:** Physico-Chemical Characterization of the Synthesized Pristine and Sulfated Zirconia Catalysts

catalyst	BET surface area (m^2^ g^–1^)	pore volume (cm^3^ g^–1^)	pore size (nm)	crystallite size^a^ (nm)	total surface acidity^b^ (mmol g^–1^)
ZrO_2_	66	0.146	8.9	8.5	0.085
0.1 SZ	137	0.099	2.8	5.1	0.09
0.5 SZ	150.4	0.114	3.0	4.6	0.16
1 SZ	187	0.13	2.9	1.1	0.19

a Crystallite size calculated by the Scherrer equation.

b Total surface acidity calculated by the titration method.

**Figure 4:**
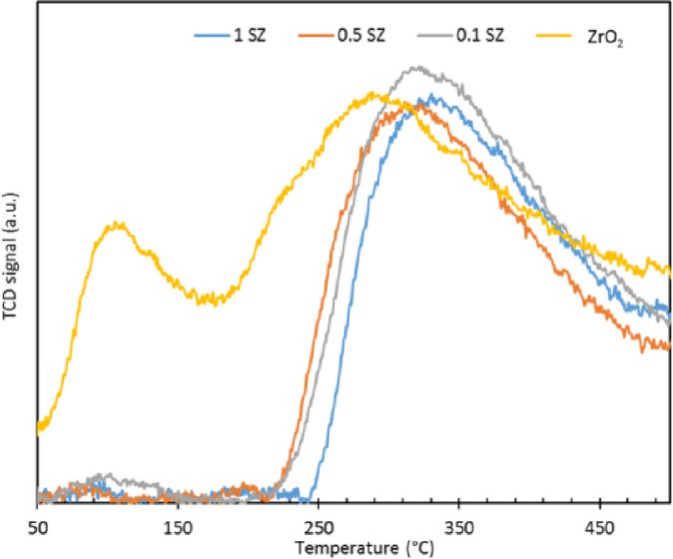
CO_2_ TPD analysis of pristine ZrO_2_, 0.1, 0.5, and 1 SZ.

The surface acidity of all catalysts was measured using the titration method reported earlier.^17^ The number of acid sites for all of the catalysts is shown in Table 2. The surface acidity of the zirconia catalysts increased linearly with the increasing extent of surface sulfation. The order in increasing acidity was ZrO_2_ < 0.1 SZ < 0.5 SZ < 1 SZ. The CO_2_ TPD analysis was performed for all investigated catalysts in order to investigate the effect of sulfation on change in the concentration of basic sites. The weak to moderately basic sites on the surface of the pristine ZrO_2_ in the range of 50–250 °C were diminished post sulfation (Figure 4). This change in the surface basicity helped to improve the selectivity of acetone as the main product which is further explained in section 3.4.

Based on the experimental observations regarding the zirconia phase stability upon sulfation, we used DFT to explore further the formation of sulfated zirconia by adsorption of one and two sulfuric acid molecules over t-ZrO_2_ (101). Since the characteristic vibrational band corresponding to the formation of dimeric species (above 1400 cm^–1^) was not observed in our FT-IR experiments,^35^ only low coverage of S-species over the catalysts were considered.

### 3.2 Identification of Surface Species by DFT Methods: H_2_SO_4_ Adsorption

As mentioned previously, the experimental characterization of this material has shown that the tetragonal polymorph of zirconia was dominant after sulfation. For this reason, we considered here the most stable surface of t-ZrO_2_, the (101) facet, to investigate the formation of surface sulfate species and the chemical modification of the surface upon sulfation. Moreover, in order to mimic distinct SO_4_ coverages, one and two sulfuric acid molecules were considered, roughly corresponding to 1 and 2 SO_4_/nm^2^, respectively. Lastly, we discuss the energy profile for the possible formation of pyrosulfate under such conditions.

#### 3.2.1 t-ZrO_2_ (101) Surface

**Figure 5:**
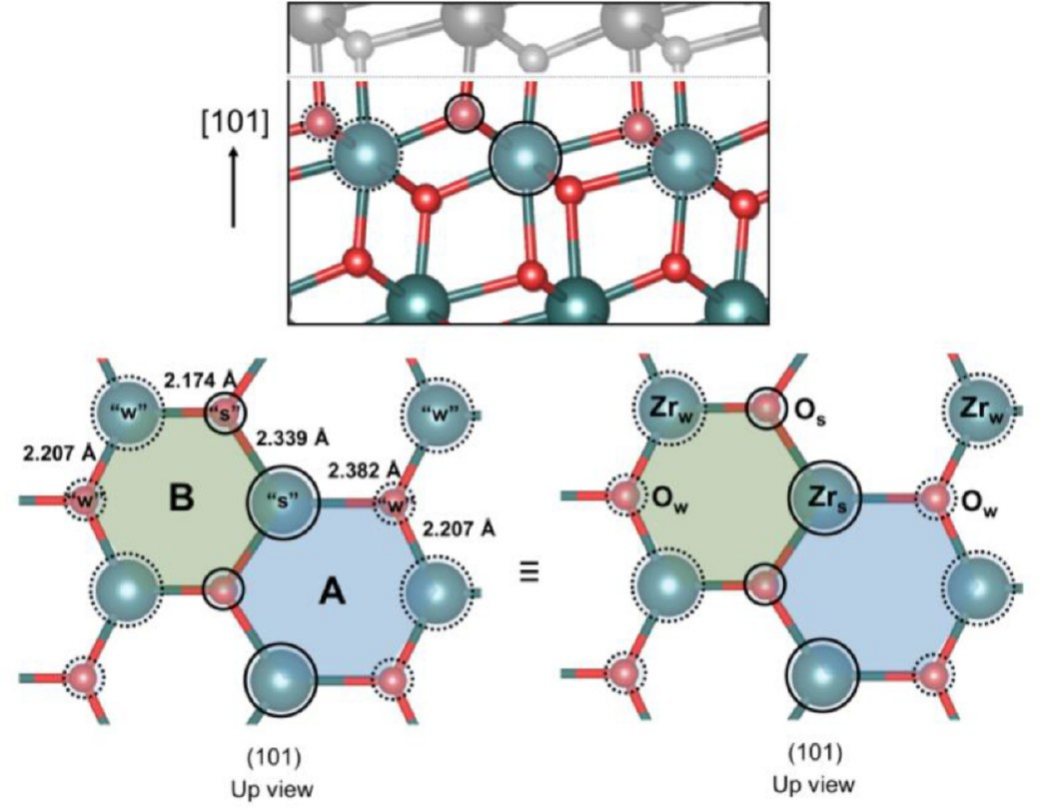
Structure of optimized t-ZrO_2_ (101) surface. Zirconium and oxygen ions are represented by gray and red balls, respectively. Dashed circles indicate weaker (“w”) acid or basic sites, whereas the solid circles indicate stronger (“s”) acid or basic sites.

The clean t-ZrO_2_ (101) is composed of two distinct types of 7-fold Zr ions and two distinct types of 3-fold O ions. As demonstrated by Haase and Sauer,^40^ when the crystal is cut along the (101) surface, two different types Zr–O bonds from bulk t-ZrO_2_ are broken, resulting in distinct types of Zr and O ions on this surface, shown in Figure 5. The so-called stronger Lewis acid site (“Zr_s_”, Figure 5) has one longer Zr–O bond of 2.382 Å and two shorter Zr–O bonds of 2.339 Å, whereas the weaker Lewis acid site (“Zr_w_”) has two longer Zr–O bonds of 2.207 Å and one shorter Zr–O bond of 2.174 Å. Similarly, the stronger (“O_s_”) basic sites are formed by one shorter and two longer bonds (2.339 and 2.174 Å) and the weaker (“O_w_”) basic site by one longer and two shorter bonds (2.382 and 2.207 Å). Interestingly, the combination of stronger and weaker sites on t-ZrO_2_ (101) leads to two distinct hexagon-shape-like tridentate absorption sites (A and B, Figure 5), which are highly relevant for the adsorption of sulfate ions over this surface, as will be discussed below.

#### 3.2.2 Sulfated t-ZrO_2_ (101) Surface: 1 H_2_SO_4_

**Figure 6:**
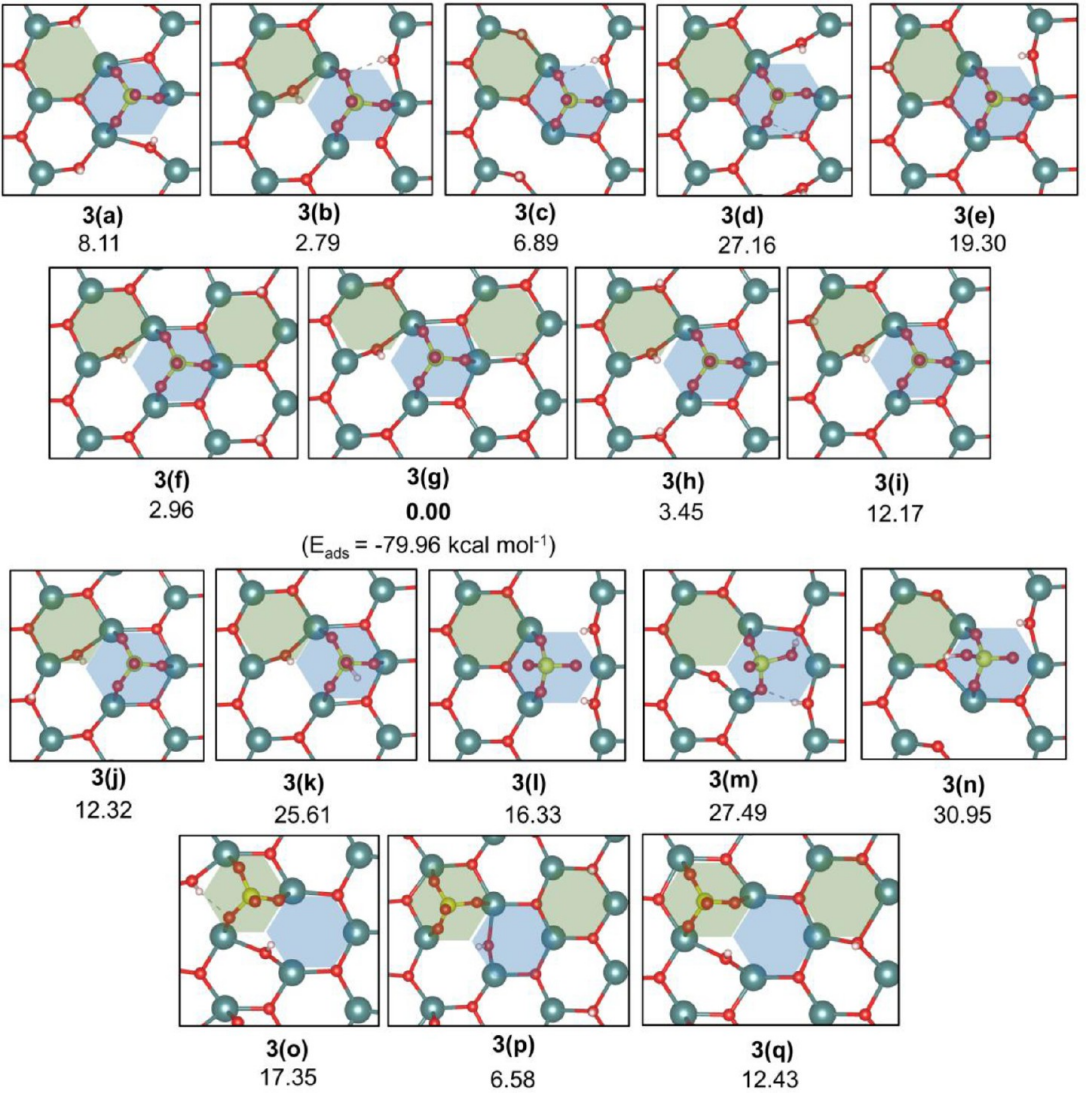
Relative energy of the most stable structures identified for H_2_SO_4_/t-ZrO_2_(101) system. Distinct adsorption sites (**a**–**n**: site “A”; **o**–**q**: site “B”), as well as alternative anchoring modes (**a**–**k**: tridentate; **l**–**n**: bidentate) and O^surf^ protonation sites (**a**–**k**) were considered. Total energy of structure **3(g)** was taken as reference to calculate the given relative energies.

In constructing the sulfated zirconia system, we initially considered the adsorption of one H_2_SO_4_ over t-ZrO_2_ (101) corresponding to a resulting coverage of approximately 1 S/nm^2^. The first step was to investigate the interaction of an H_2_SO_4_ molecule, as well as partially deprotonated HSO_4_^–^ and fully deprotonated SO_4_^2–^ groups (Figure 6), with the adsorption site “A” of t-ZrO_2_ (101). Since this site comprises two Zr_s_ (stronger) Lewis acid sites, it would be expected to result in the largest adsorption energy for sulfate groups. Neutral H_2_SO_4_ did not interact strongly with the t-ZrO_2_ (101) except when the deprotonation of S–OH groups was allowed. The adsorption of partially deprotonated HSO_4_^–^ (Figure 6, **3(k)**, **3(m)**, and **3(n)**) resulted in higher energies (i.e., less exothermic) than those obtained for the adsorption of SO_4_^2–^ groups. Finally, the most stable configurations contained SO_4_^2–^ groups anchored in the tridentate fashion, with their protons either at O_s_ sites only (Figure 6, **3(f)** and **3(g)**) or at both O_s_ and O_w_ sites (Figure 6, **3(b)**). The calculated adsorption energy for **3(g)** was −79.96 kcal mol^–1^.

Using the most stable structures identified for the dissociative adsorption of H_2_SO_4_ on site “A” of the t-ZrO_2_ (101) surface (illustrated in Figure 6, **3(b)**, **3(f)**, and **3(g)**), analogous structures were used to determine the energy differences when site “B” is considered. As shown in Figure 6 (**3o**–**q**), the energy difference from **3(g)** varied between 6.58 and 17.35 kcal mol^–1^, in agreement with our initial assumption that adsorption of sulfate groups at site “A” should be favored due to the presence of two Zr_s_ (stronger) Lewis acid sites. The calculated adsorption energies for all investigated cases can be found in the Supporting Information (Table S1).

#### 3.2.3 Sulfated t-ZrO_2_ (101) Surface: 2 H_2_SO_4_

**Figure 7:**
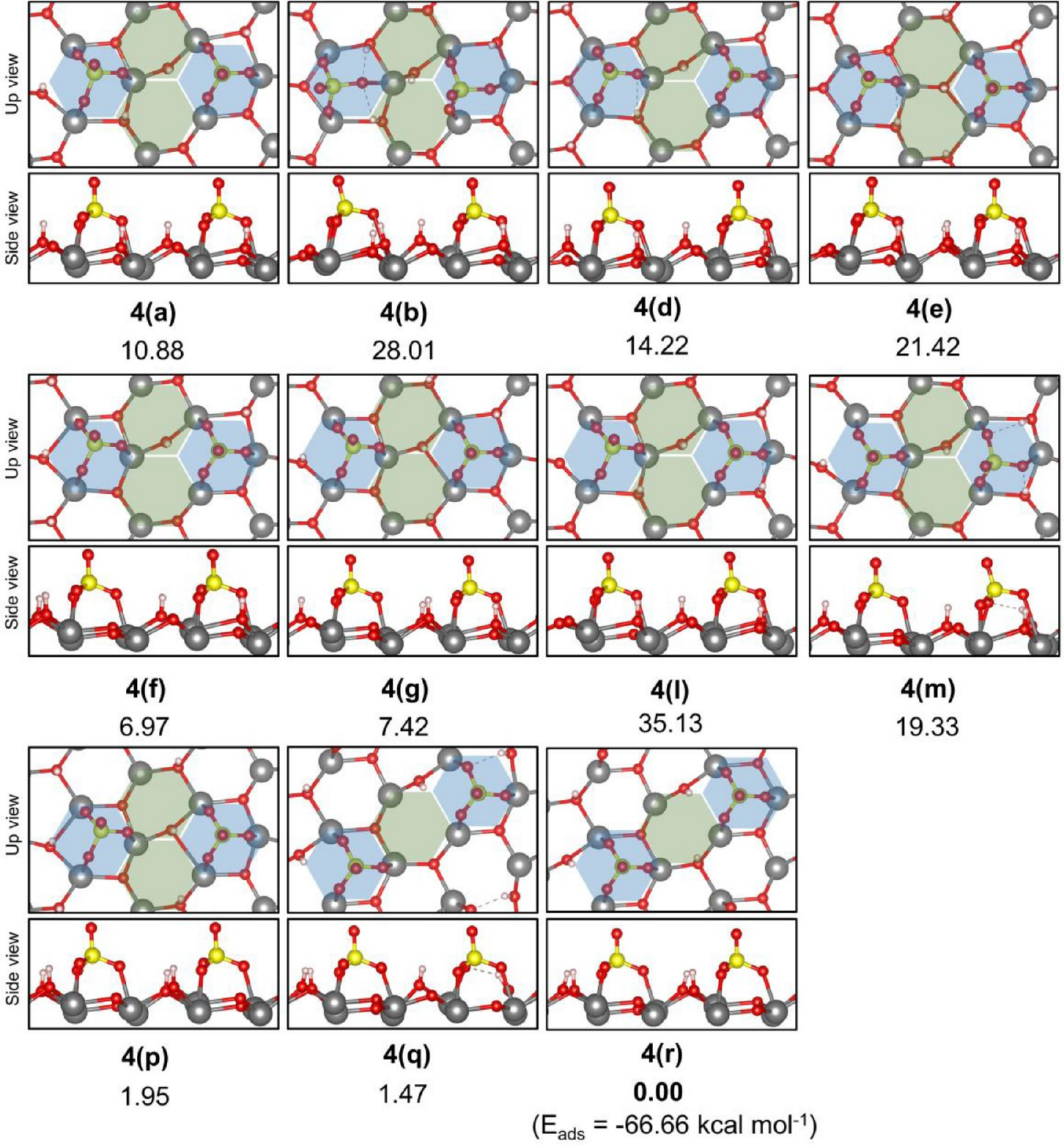
Relative energy of the most stable structures identified for dissociate adsorption of a 2nd H_2_SO_4_ at adsorption site “A” (blue hexagon) of structure **3(g)**. Alternative alignment of adsorption site “A” in [110] (**a**–**p**) and [010] (**q** and **r**) directions where considered. Total energy of structure **4(r)** was taken as the reference to calculate the given relative energies.

In order to investigate higher SO_4_^2–^ coverages (approximately, 2 S/nm^2^), we modeled the adsorption of a second H_2_SO_4_ molecule over structure **3(b)** (Figure 6). Again, distinct adsorption sites “A” and “B” were considered, as demonstrated in Figures 6 and 7, respectively. For all cases investigated, the initial configurations were adjusted to provide a tridentate-like adsorption of the second SO_4_^2–^ group, similar to that calculated for the most stable structures of a single SO_4_^2–^ on the zirconia surface. Additionally, alternative protonated O^surf^ sites were examined in detail. The adsorption of SO_4_^2–^ at distinct “A” sites of the t-ZrO_2_ (101) surface (Figure 7) resulted in well-behaved structures after optimization, presenting tridentate sulfate groups with only small changes of the surface bond lengths due to the protonation of distinct basic sites. The most stable cases were those where the four protons from the two sulfuric acid molecules were captured by the stronger surface basic sites (O_s_). Interestingly, an alignment of sulfate groups in the [010] direction (Figure 7, **4(r)**) was observed to be more favorable than that in the [110] direction.

**Figure 8:**
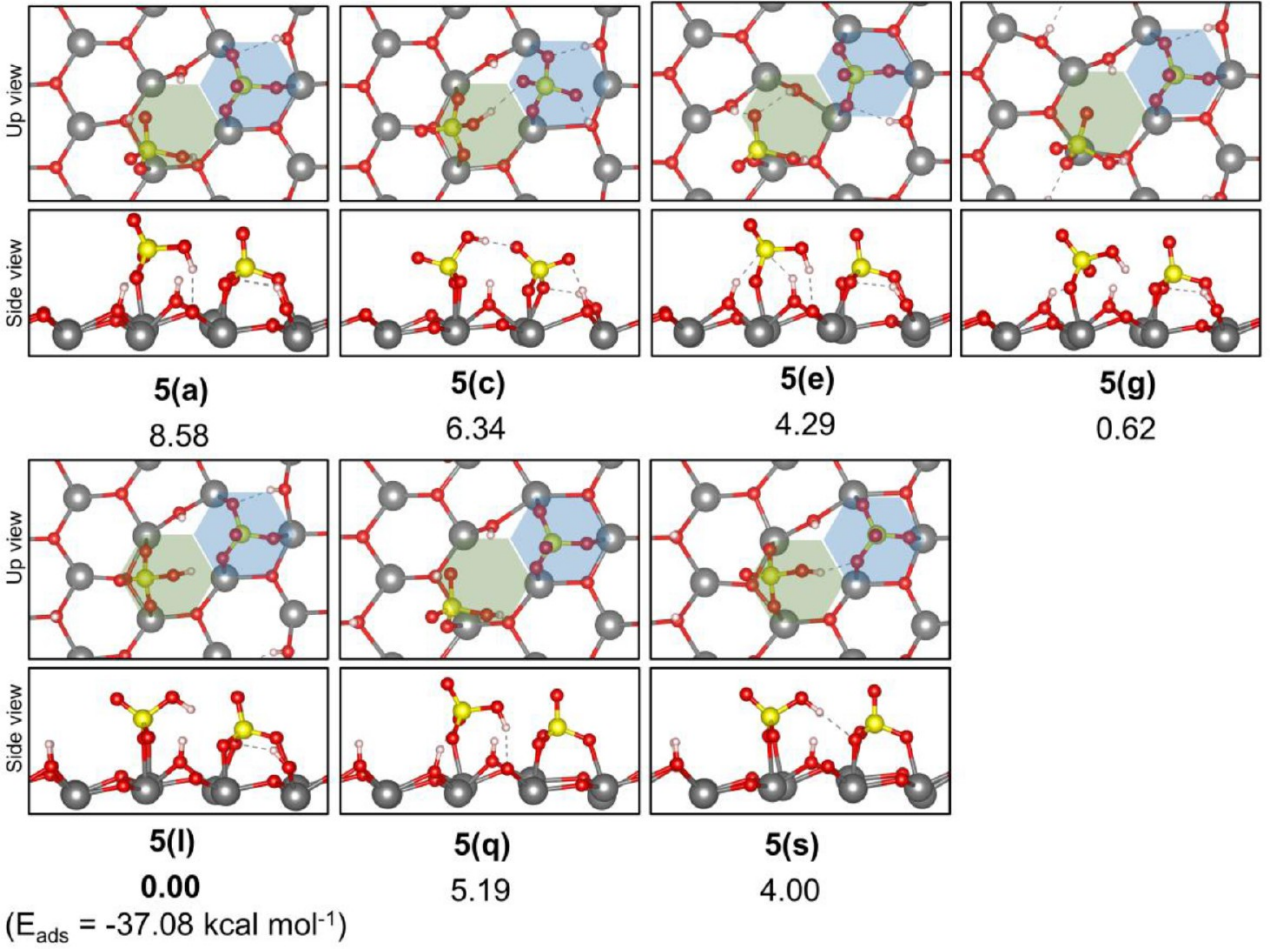
Relative energy of the most stable structures identified for dissociate adsorption of a 2nd H_2_SO_4_ at adsorption site “B” (green hexagon) of structure **3(g)**. The total energy of structure **5(l)** was taken as a reference to calculate the given relative energies. The full list of tested structures can be found in the Supporting Information (Table S2).

The adsorption of a second sulfate group at the “B” site resulted in a more complex behavior. The most stable cases are presented in Figure 8 and Table S2 (Supporting Information). We found that the anchoring mode of both SO_4_^2–^ groups in such systems was highly dependent on which O^surf^ sites were protonated. For structures **5a**,**c**,**e**–**s**, the sulfate group at the “A” sites remains adsorbed in a tridentate fashion, whereas that at “B” site abstracted one of the protons from the surface to form an HSO_4_^–^ specie, which interacted with both the surface and neighboring sulfate group, or, solely with surface sites. While the interaction with the neighboring sulfate group occurred via an S–OH···O–S hydrogen bond, its interaction with the surface involved both adsorption at Zr sites and an S–OH···O^surf^ hydrogen bond. Interestingly, despite the significant changes observed in the structure of these systems, their relative energies varied no more than 8.6 kcal mol^–1^. We note, however, that the computed adsorption energies for these cases (e.g., −37.08 kcal mol^–1^ for **5(l)**) are much smaller than those calculated for the adsorption of the second sulfate group at an “A” site (−66.66 kcal mol^–1^ for **4(r)**). These results indicate that site “B” will probably be occupied only at higher sulfate coverages or only to form a transient intermediate structure for the formation of dimeric species on the surface. It is worth noting that two sulfate groups at “A” sites would not be able to interact to form the pyrosulfate specie and that the migration of one SO_4_^2–^ to site “B” would be required for such a reaction to occur.

The identity of sulfur-containing species over zirconia for different SO_4_^2–^ coverages and experimental conditions has been intensively debated over the last decades. One of the widely accepted hypotheses is that surface pyrosulfates form at higher coverages (4 S/nm^2^) and are responsible for the observed catalytic activity of SZ toward the isomerization of alkanes.^1^ The inequivalent character of the two S atoms in such a dimer and the experimentally observed vibrational band above 1400 cm^–1^ have been extensively used to justify this assumption. However, as shown here, upon adsorption of SO_4_^2–^ anions at both “A” and “B” sites, the formation of HSO_4_^–^ is spontaneously observed during structure optimization through the abstraction of one proton from a surface O site. These species (HSO_4_^–^ at “B” and SO_4_^2–^ at “A”) are also expected to present chemically distinct S species and vibrational frequencies at 1412 or 1474 cm^–1^, as previously reported.^40,43^ Interestingly, the energy profile for the dimerization of adsorbed sulfates has not been considered in previous works and such an investigation can be key to the understanding of the proposed condensation reaction or to verify the feasibility of hydrolysis under water washing conditions at ambient conditions, as performed by Li et al.^34^ and suggested by Breitkopf and co-workers.^45^ To this aim, we computed the energy barriers for the possible formation of surface pyrosulfate species, as discussed next.

#### 3.2.4 Pyrosulfate Formation

**Figure 9:**
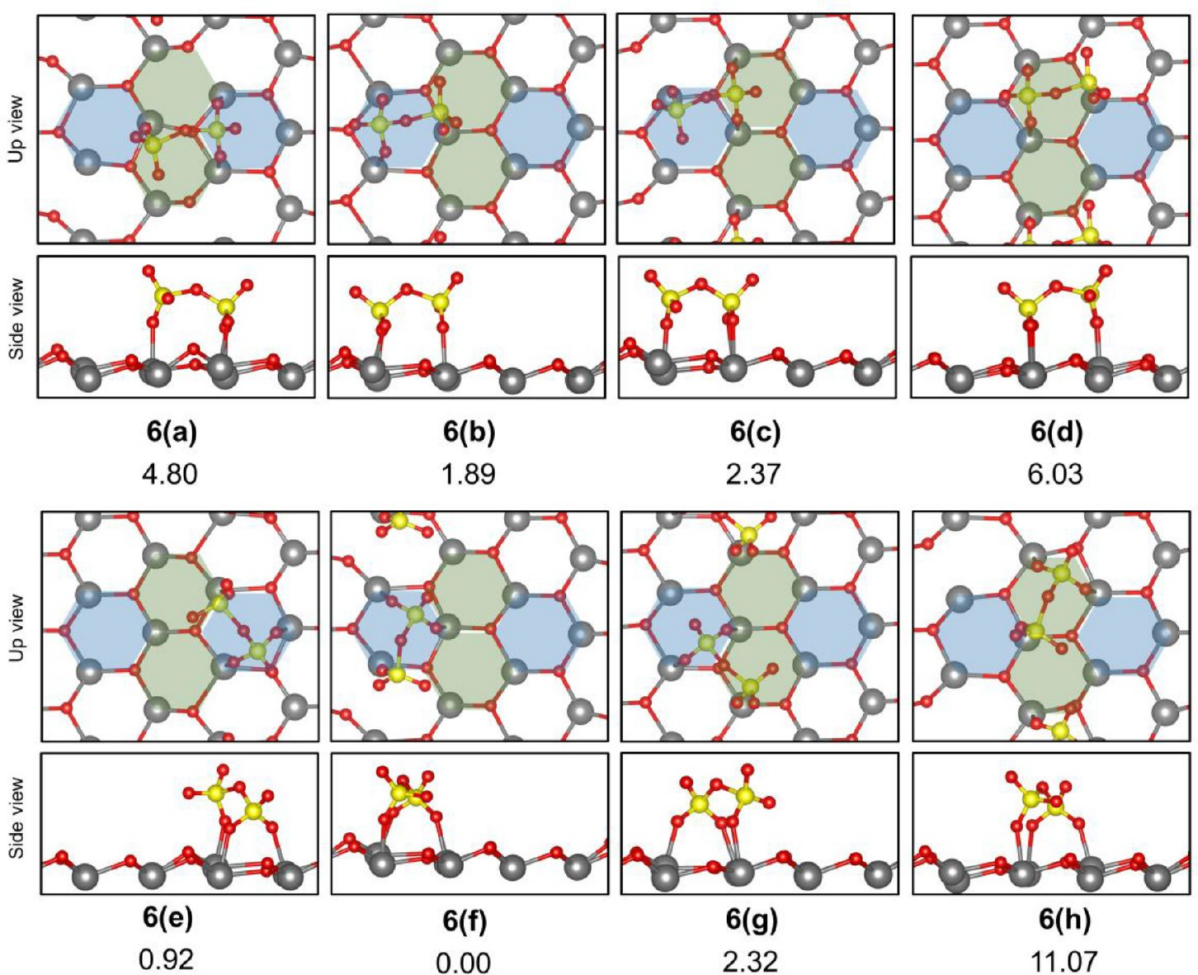
Relative energy of the most stable structures identified for adsorption of S_2_O_7_^2–^ anion over t-ZrO_2_ (101). Total energy of structure **6(f)** was taken as the reference to calculate the given relative energies.

Initially, alternative adsorption modes of the pyrosulfate anion (S_2_O_7_^2–^) were investigated over the t-ZrO_2_ (101) surface (Figure 9), followed by the comparison of alternative protonated O^surf^ sites (Figure S2, Supporting Information). Interaction of the pyrosulfate anion (S_2_O_7_^2–^) with the t-ZrO_2_ (101) surface was observed also to occur in a tridentate fashion, as shown in Figure 9. For this case, the interaction with the surface acid sites can be observed at a single “A” or “B” site (Figure 9, **6b**,**d**,**e**,**f**,**h)**) or at both sites, simultaneously (Figure 9 (**6a**,**c**,**g)**). The most stable case corresponded to one S–O group from each O–SO_3_ monomer of pyrosulfate allowed to interact with a Zr_s_ site, while a third S–O interacted with a Zr_w_ (Figure 9, **6(f)**). As expected, the comparison of alternative protonated O^surf^ sites (**Figure S2**, Supporting Information) showed that protonated O_s_ basic sites resulted in the most stable structures, as shown for **7(e)** (Figure S2, Supporting Information).

**Figure 10:**
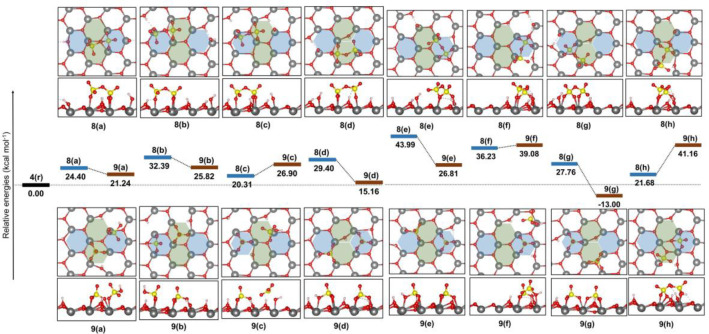
Relative energy of the most stable structures identified for initial (**9a**–**9h**) and final (**8a**–**8h**) reactive complexes ([H_2_S_2_O_7_ + H_2_O] and [2 H_2_SO_4_]) for the pyrosulfate formation over t-ZrO_2_ (101). Total energy of structure **4(r)** was taken as reference to calculate the given relative energies.

In general, all alternative structures considered (Figure 9) resulted in minor energy differences, which seems to indicate that all structures could be energetically accessible under experimental conditions. This result is important when considering the alternative reaction pathways in forming pyrosulfate from the isolated sulfate species, since significant changes in the energy barriers for the process may be observed between these cases. For this reason, eight putative reaction paths were investigated, as well as the corresponding initial and final reactive complexes (Figure 10) were considered for the formation of the adsorbed pyrosulfates described in Figure 9 from the appropriately adsorbed sulfate anions.

All dimeric systems [S_2_O_7_^2–^,2H^+^,H_2_O] obtained in this investigation were calculated to have a higher energy than those of the monomeric reference system **4(r)**, with computed relative energies varying between 20.31 and 43.99 kcal mol^–1^. These results indicate that formation of dimeric species would only be favored under harsh/dehydrating experimental conditions and, possibly, only at higher coverages than those investigated here. Under mild and water-rich conditions, the hydrolysis of the dimeric species (if present, at all) would be favored to produce isolated SO_4_^2–^ on the surface of the catalyst. Moreover, the transition state structures for four of the eight investigated reaction paths were obtained and they revealed that the activation energy for the dimerization process ranges between 60.0 and 70.0 kcal mol^–1^. Such high energy barrier to form pyrosulfates on the zirconia surface is unlikely to be surmounted even under calcinating conditions. Taking structure **4(r)** as reference, the calculated energy barrier for each of these cases were as follows: **8(a)** → **9(a)**, *E*^‡^ = 68.9 kcal mol^–1^; **8(b)** → **9(b)**, *E*^‡^ = 61.0 kcal mol^–1^; **8(d)** → **9(d)**, *E*^‡^ = 60.2 kcal mol^–1^; *E*^‡^ = **8(f)** → **9(f)**, 70.3 kcal mol^–1^. These results agree with the finding by Sauer et al., who suggested that the experimental conditions will determine the surface species formed over zirconia: high SO_4_^2–^ concentrations, high calcination temperatures, and dehydration conditions favor dimeric species, whereas low SO_4_^2–^ concentrations, low calcination temperatures, and hydration conditions will favor the monomeric system. It is worth noting that higher order polymeric systems were found to be unlikely to be formed.^40,43^

Based on these observations, the investigation of changes in acidity and basicity of sulfated zirconia investigated in this work were mapped by DFT methods (section 3.3) considering only the monomeric system (structures **3b** and **3g**, Figure 6). Although this choice may differ from that used by Sauer for investigation of the isomerization of alkanes,^35,45^ this is due to the distinct composition of the catalysts used here (lower S coverages), as well as the experimental conditions later employed for studying ketonization reactions in biofuel upgrading (mild and water-rich conditions).

### 3.3 Acid–Base Properties of Pristine and Sulfated Zirconia

As discussed in section 3.1, sulfation of zirconia led to a linear increase of its acidity (as determined by the titration method) and CO_2_ TPD analysis revealed a concomitant reduction of the number of basic sites in the catalyst. In order to provide a more detailed description of the distinct acid and basic sites on t-ZrO_2_ (101) and the corresponding sulfated surface (structures **3b** and **3g**, Figure 6), DFT calculations were also employed to investigate the adsorption of probe molecules (pyridine, NH_3_, CO_2_, and H_2_O) over the modeled surfaces. H_2_O dissociative adsorption was also used for pristine zirconia as a source of surface protons for subsequent estimation of its Brønsted acidity by computation of the corresponding proton transfer energies.

**Figure 11:**
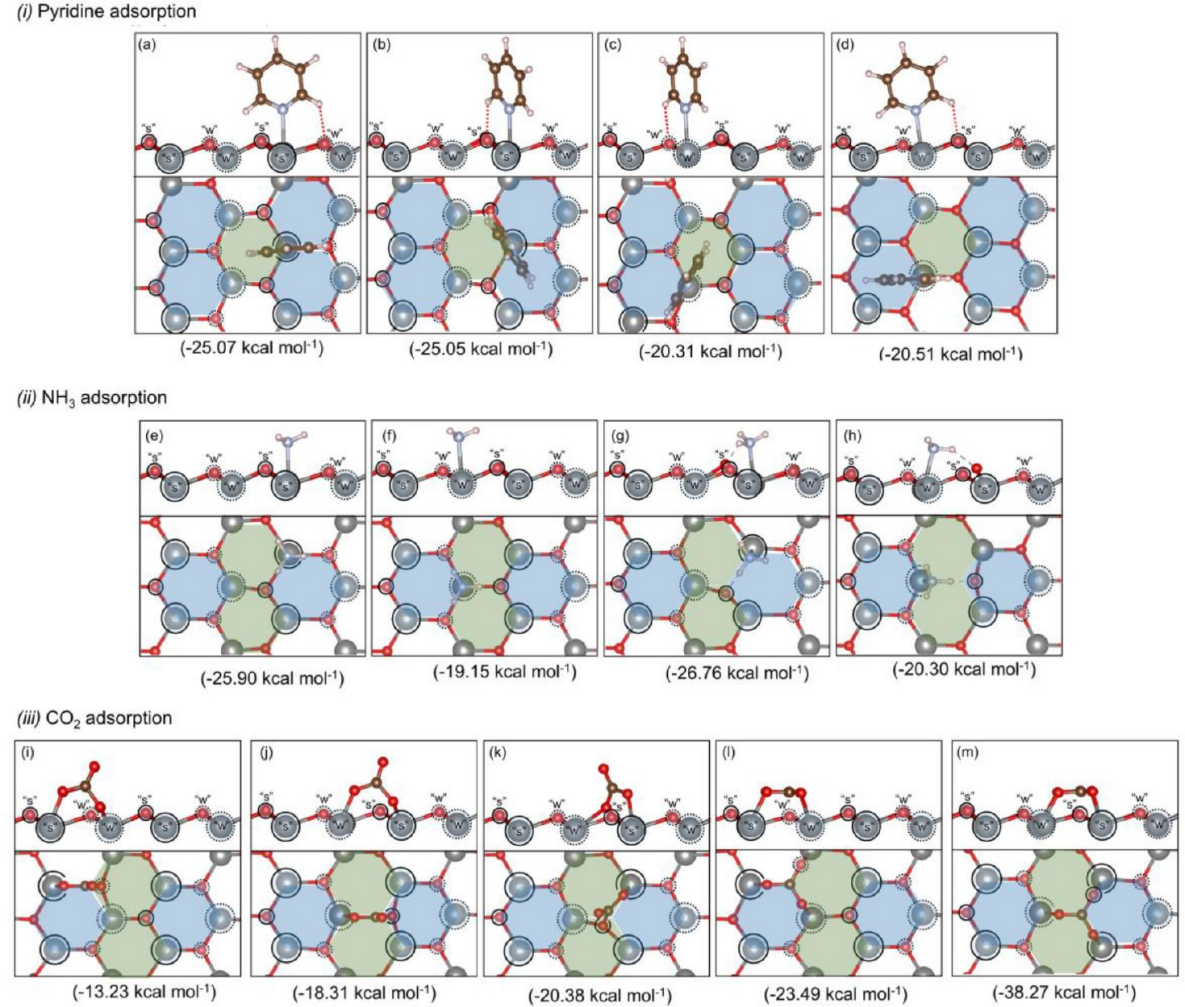
Distinct adsorption modes and calculated adsorption energy for pyridine (a–d), NH_3_ (e–h), and CO_2_ (i–m) over t-ZrO_2_ (101).

After adsorption of pyridine over t-ZrO_2_ (101), two important interactions were observed between the adsorbate and the surface: first, the lone electron pair of nitrogen interacting with the Zr site and, second, the weaker polar interaction between the CH group at the ortho position of the aromatic ring of the pyridine and the O sites (see Figure 11). As a result, four adsorption energies could be calculated. The stronger acid site Zr_s_ gave adsorption energies for pyridine of circa −25 kcal mol^–1^ (as shown in Figure 11a,b), whereas Zr_w_ resulted in smaller adsorption energies by roughly 5 kcal mol^–1^ (Figure 11c,d). Only very small energy differences were observed for configurations with different interactions between the pyridine ortho CH group and sites O_s_/O_w_. The adsorption of NH_3_ was investigated, first by placing the molecule simply on top of Zr_s_ or Zr_w_ acid sites and, second, by allowing additional NH···O^surf^ interactions, where O^surf^ is any surface oxygen (either O_s_ or O_w_ sites). For the first case (top position), the adsorption energies were −25.90 and −19.15 kcal mol^–1^ (Figure 11e,f), for Zr_s_ or Zr_w_, respectively. When the H-bond interaction between the absorbate and surface was considered (sites Zr_s_-O_s_ and Zr_w_-O_s_, Figure 11g,h), additional stabilization of the system of approximately 1 kcal mol^–1^ was observed. Interestingly, such a NH···O^surf^ interaction was only observed for O_s_; when O_w_ was considered, the structure optimization resulted in the migration of the substrate back to the top position of sites Zr_s_ and Zr_w_.

For the adsorption of CO_2_ over the clean t-ZrO_2_ (101) surface, two distinct types of adsorption modes were observed: (i) CO_2_ activation by surface oxygen and adsorption of the activated CO_2_ perpendicular to the surface (η^2^-CO_2_ adsorption mode, Figure 11i–k) and (ii) CO_2_ activation by surface oxygen, followed by migration of the same surface oxygen away from the surface, resulting in the adsorption of the activated CO_2_ (or CO_3_^2–^) parallel to the surface (η^3^-CO_2_ adsorption mode, Figure 11l,m). Both adsorption modes are relevant here and will be key for mapping the changes in the reactivity of the catalysts, not only to evaluate the basicity of oxygen sites, but also the interplay between acid-basic sites (in η^2^-CO_2_ adsorption) and changes in the trends of oxygen vacancy formation (in η^3^-CO_2_ adsorption). For the first case (η^2^-CO_2_), three distinct adsorption energies were calculated, −13.23, −18.31, and −20.38 kcal mol^–1^ for O_w_-Zr_s_, O_s_-Zr_w_, and O_s_-Zr_s_, respectively (Figure 11i–k). Interestingly, CO_2_ adsorption/activation was not observed for O_w_ when the auxiliary C–O···Zr_w_ was investigated. For the second type of CO_2_ activation/adsorption (η^3^-CO_2_), despite the required abstraction of one surface oxygen by CO_2_ to form η^3^-CO_2_, such structures gave the largest adsorption energies, with values of −23.49 and −38.57 for O_w_ and O_s_, respectively (Figure 11l,m).

**Figure 12:**
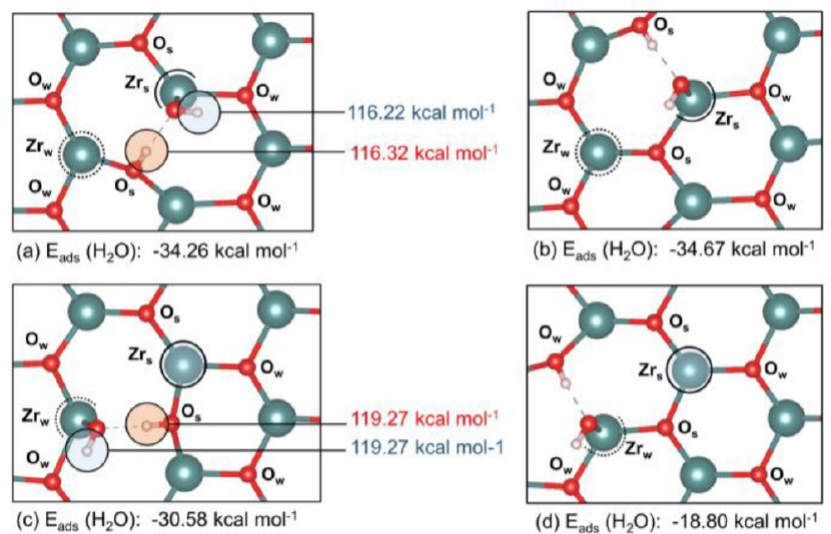
Adsorption energy of one H_2_O molecule on t-ZrO_2_ (101) at Zr_s_ (a and b) and Zr_w_ (c and d) acid sites. Calculated proton transfer energies of O^surf^–H and O^top^–H groups formed after dissociative adsorption of H_2_O are shown in red and blue values, respectively. Optimization after deprotonation of O^top^–H led to abstraction of the proton in O^surf^–H by the remaining O^top^.

Finally, dissociative adsorption was modeled for H_2_O at all four inequivalent adsorption configurations over t-ZrO_2_ (101) (a–d, Figure 12), considering Zr_s_ and Zr_w_ acid sites and auxiliary HO-H···O_w_ and HO–H···O_s_ interactions. For the cases a–c (Figure 12), the optimization process led to spontaneous abstraction of one proton from H_2_O by an O_s_ surface site and gave the most stable structures with adsorption energies varying between ∼−30 and −35 kcal mol^–1^. The abstraction of a proton by an O_w_ site occurred only when O–H···O_w_ was the remaining auxiliary interaction, as shown in Figure 12d. In this case, the calculated adsorption energy (−18.80 kcal mol^–1^) was much lower than those calculated before.

The Brønsted acidity was estimated by calculating the proton transfer energy from a protonated surface site to a water molecule located in the vacuum between slabs. The deprotonation of the H_2_O/t-ZrO_2_ (101) system was evaluated considering two distinct types of protons on the surface, according to the deprotonation equation below (eq 3). The first proton considered was that abstracted from the adsorbed H_2_O by a surface oxygen site (O^surf^-H^+^) and the second proton was that at the adsorbed hydroxyl group (*OH^–^) (Figure 12). For the latter, structure optimization of the deprotonated system led to the remaining *O group to recover a proton from O^surf^-H^+^, restoring the original *OH^–^ group.
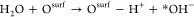
3

As performed for the clean surface, we also employed pyridine, NH_3_, and CO_2_ molecules to probe the changes in acidity and basicity of the sulfated t-ZrO_2_ (101) surface sites. For this, we used the most stable structure previously identified (**3(b)** and **3(g)**, Figure 6), considering all inequivalent acid and basic sites in a 2 × 2 t-ZrO_2_ (101) surface: eight Zr ions and eight O ions (Tables 3–6). Vibrational frequency calculations for the most stable CO_2_- and pyridine-containing systems identified below are reported in the Supporting Information (Tables S3–S6).

**Table 3:**
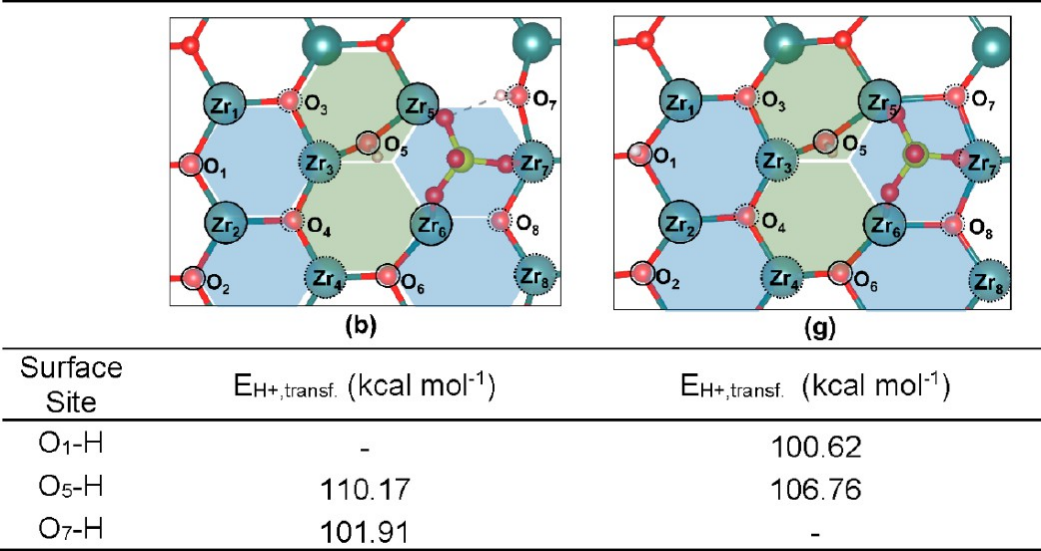
Calculated Proton Transfer Energies (*E*_H+,transf_.) for Inequivalent Protons on Structures **3(b)** and **3(g)**

First, we considered the calculated proton transfer energy for the protonated surface sites O_1_–H, O_5_–H, and O_7_–H (Table 3), which were significantly smaller than those calculated for the deprotonation of H_2_O on the pristine t-ZrO_2_ (101) surface (Figure 12). Two very distinct Brønsted acid sites can be expected in such structures, as the energy differences between O_1_–H (or O_7_–H) and O_5_–H varied between 8.26 and 6.14 kcal mol^–1^, in agreement with previous experimental observations.^57^

The lowest proton transfer energy values were obtained for O_1_–H and O_7_–H (100.62 and 101.91 kcal mol^–1^, respectively). Such a drop in the calculated proton transfer energies (or, conversely, increase of Brønsted acidity of the surface) if compared to those calculated for the H_2_O/t-ZrO_2_ (101) system, can be rationalized in terms of the inductive electron withdrawing effect imposed by the adsorbed of SO_4_^–2^ anion over the surface.^58−60^ The greater acidity of the surface is consistent with the results obtained for pyridine and NH_3_ adsorption (Tables 5 and 6), as will be discussed next.

When pyridine adsorption was considered (Table 4), surface Zr_s_ sites Zr_1_ and Zr_2_ gave adsorption energies ranging from −27.15 to −30.97 kcal mol^–1^, whereas Zr_w_ sites led to values ranging between −15.65 and −18.33 kcal mol^–1^ for Zr_3_, −23.34 and −26.83 for Zr_4_ kcal mol^–1^, and −21.87 and −28.56 kcal mol^–1^ for Zr_8_. In all these cases, it was possible to observe a significant increase in the acidity of both Zr_s_ and Zr_w_ sites when compared to the clean surface (Figure 11), in agreement with previous experimental work.^61,62^ However, a reduction of the available Zr sites was also observed, as now three of the eight surface Zr ions were occupied by the adsorbed SO_4_^–2^ anion (Zr_5_, Zr_6_, and Zr_7_). Interestingly, attempts to force the pyridine adsorption at Zr_5_, Zr_6_, and Zr_7_ site, which could result in displacement of the SO_4_^–2^ group, as discussed by other authors,^41^ led to desorption or less exothermic adsorption energies. Similar results were obtained when NH_3_ adsorption was considered (Table 5), except for NH_3_ adsorption at Zr_5_ and Zr_6_ sites of structure **3(g)**, which were the only cases where SO_4_^–2^ displacement was observed. Due to the small energy differences detected for the clean surface when considering adsorption of NH_3_ at the top position or when considering additional H-bond interaction between the absorbate and the surface (Figure 11), only the former was considered here.

**Table 4:**
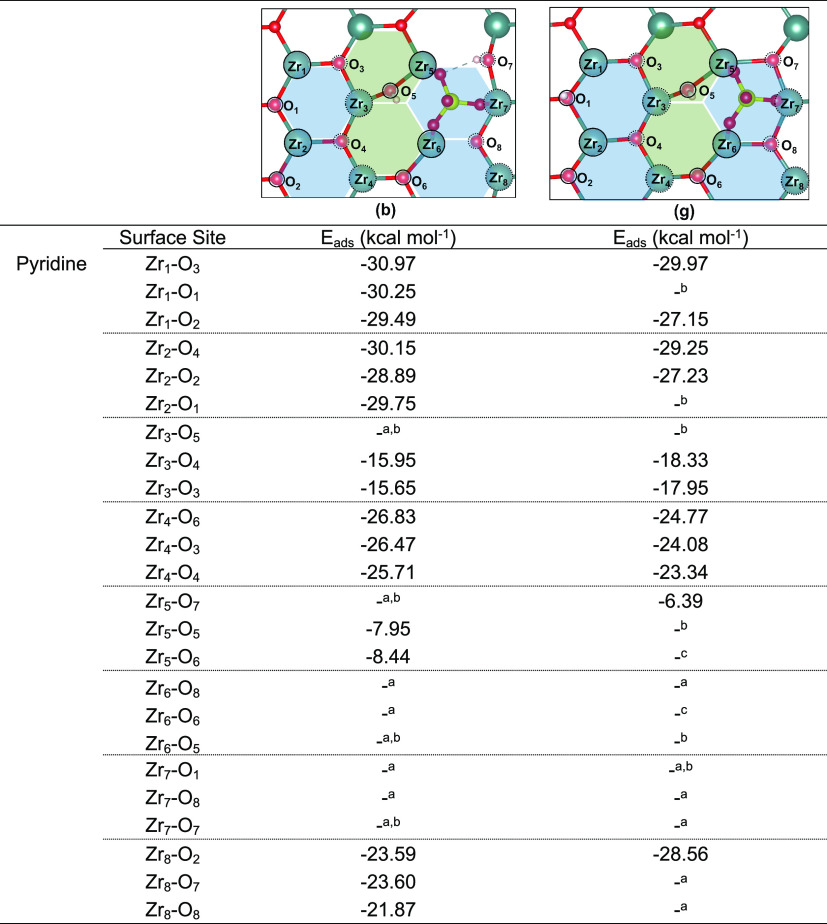
Calculated Adsorption Energies for Pyridine over Inequivalent Acid Sites of Structures **3(b)** and **3(g)**

^*a*^ Desorption was observed upon structure optimization. ^*b*^ Protonated Osurf site. ^*c*^ Zr–O–S site.

**Table 5:**
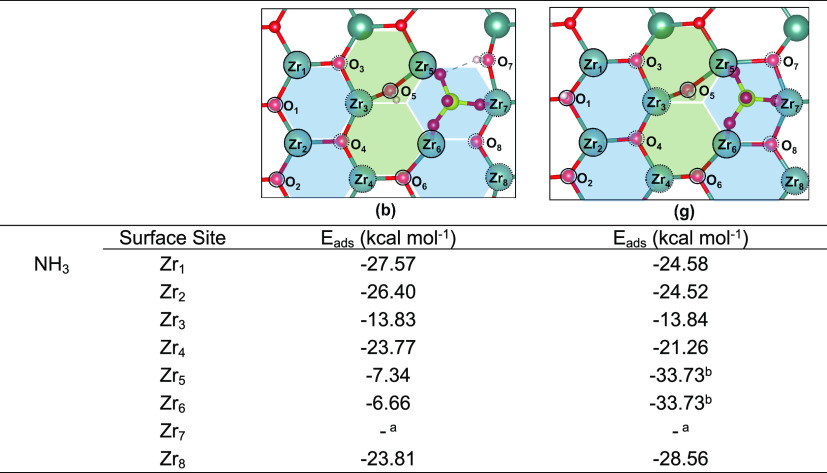
Calculated Adsorption Energies for NH_3_ over Inequivalent Acid Sites of Structures **3(b)** and **3(g)**

^*a*^ Desorption was observed. ^*b*^ NH_3_ led to displacement of adsorbed sulfate.

In general, both the availability and strength of the Lewis basic sites were significantly reduced upon sulfation. First considering the η^2^-CO_2_ adsorption mode (Table 6), two O_s_ surface sites (O_1_ and O_2_) in **3(b)** gave computed adsorption energies between −8.26 and −12.41 kcal mol^–1^, whereas the other O_s_ surface site present in the investigated surface, O_6_, presented positive adsorption energies. Adsorption at the O_w_ sites gave positive (or only slightly negative) adsorption energy values (see adsorption at O_3_ site, Table 6) or showed desorption of the absorbed CO_2_ upon structure optimization (see O_4_ and O_8_, Table 6). When the η^3^-CO_2_ adsorption mode was considered, the O_s_ surface sites, O_1_ and O_2_ resulted in computed adsorption energies of −16.64 and −24.74 kcal mol^–1^, whereas the O_w_ surface site O_3_ and O_4_ resulted in much smaller adsorption energies, −6.59 and −6.68 kcal mol^–1^, respectively. For O_6_ and O_8_, η^3^-CO_2_ adsorption was not observed and optimization of such structures always led to η^2^-CO_2_ adsorption mode. In all cases, the computed adsorption energies of CO_2_ over the sulfated surface were significantly smaller than those obtained for the clean t-ZrO_2_ (101) surface (Figure 6). Similar behavior was observed for structure **3(g)**. These results are in agreement with observations from CO_2_ TPD analysis (section 3.1) and indicate that the disappearance of weaker CO_2_ adsorption modes may be attributed to the very low basicity of O_w_ surface sites after sulfation, whereas O_s_ surface site remain active toward CO_2_ capture, although with slightly small adsorption energies.

**Table 6:**
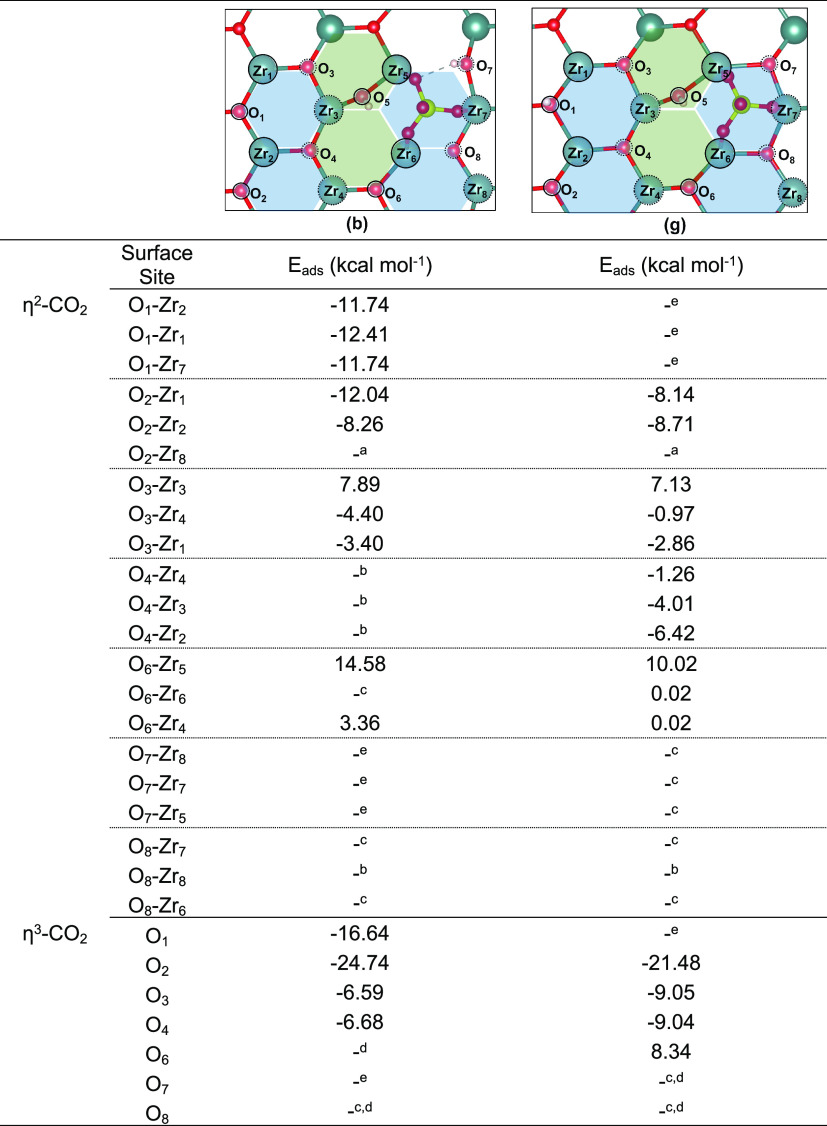
Calculated Adsorption Energies for CO_2_ over Inequivalent Basic Sites of Structures **3(b)** and **3(g)**

*^a^* Only η^3^-CO_2_ configuration was obtained. ^*b*^ Desorption was observed upon structure optimization. *^c^* Zr–O–S site; ^*d*^ Only η^2^-CO_2_ configuration was obtained. *^e^* Protonated O site.

Overall, theoretical and experimental results showed an increase of the acidity and reduction of the strength and number of available basic sites of the zirconia catalyst upon sulfation. The effect of such modifications over its catalytic activity, however, depends on the interplay between them, as the ketonization process requires both acidity and basicity of the active sites to be tuned. In the last stage of this work, the effect of such changes was explored in the ketonization of acetic acid.

### 3.4 Ketonization of Acetic Acid: Influence of Sulfation, Increasing Acidity, and Decreasing Basicity on the Catalyst Surface

**Figure 13:**
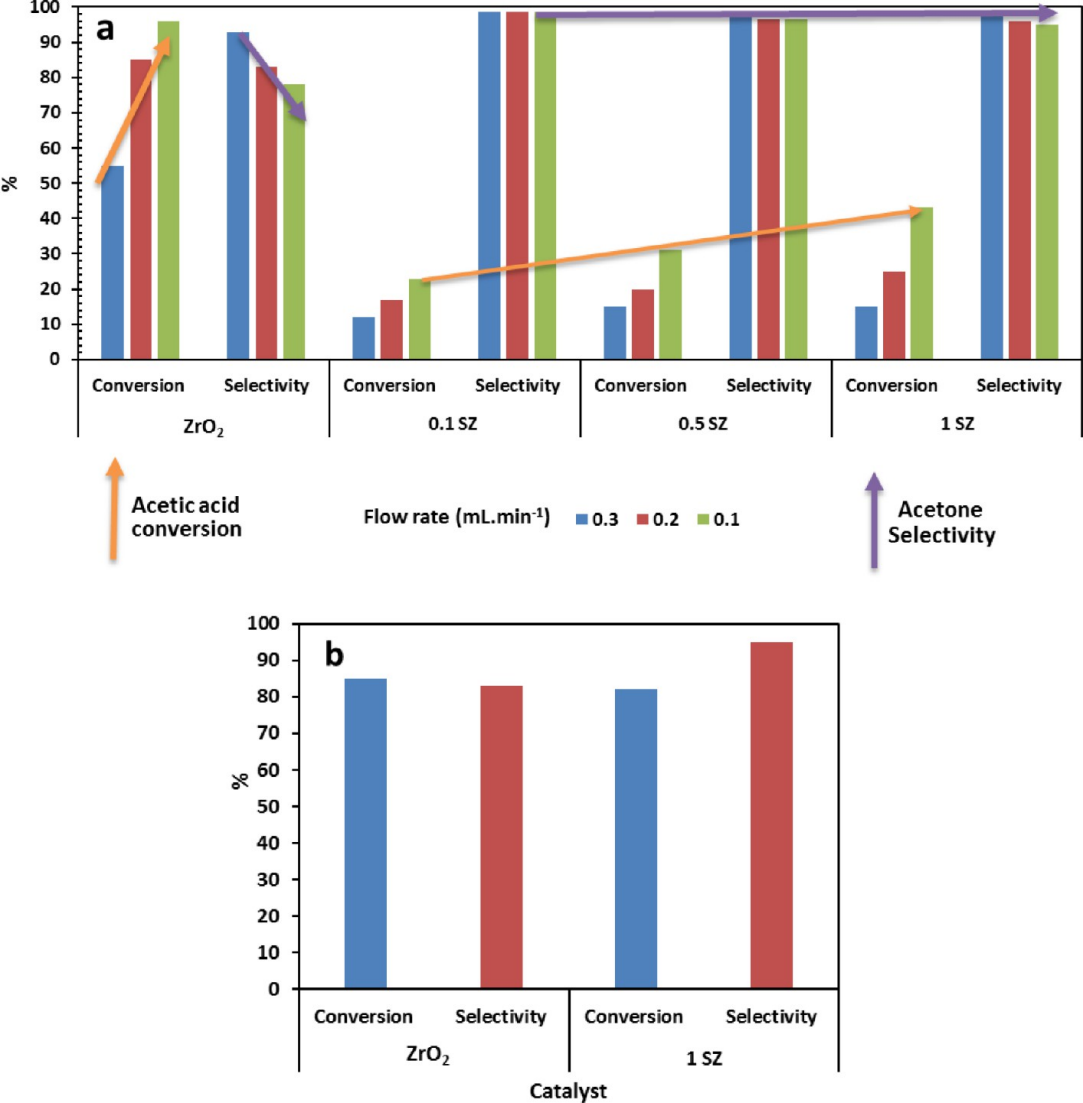
(a) Ketonization of acetic acid to acetone over ZrO_2_ and sulfated ZrO_2_ catalysts with increasing sulfate ion coverage at varying feed flow rates 0.3, 0.2, and 0.1 mL min^–1^. (b) Comparison of pristine ZrO_2_ and 1 M ZrO_2_*. Reaction conditions: catalyst loading, 2 g; reaction temperature, 350 °C; *catalyst loading, 4 g; and feed flow rate, 0.01 mL min^–1^.

**Figure 14:**
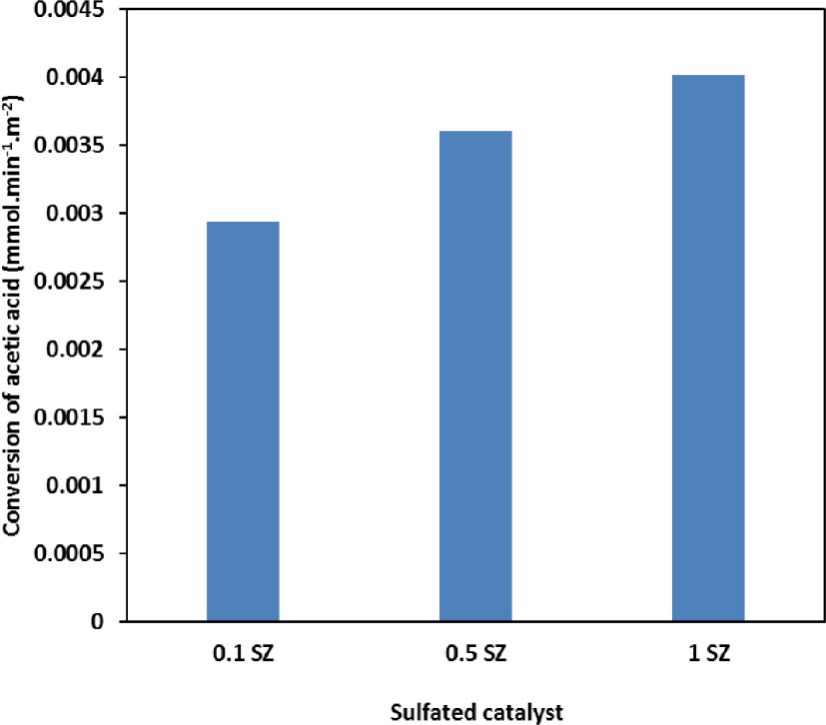
Comparison of normalized conversion of acetic acid for sulfated zirconia catalysts. Reaction conditions: feed flow rate, 0.1 mL min^–1^; reaction temperature, 350 °C; catalyst loading, 2 g.

The activity of the catalysts (ZrO_2_, 0.1, 0.5, and 1 SZ) was studied in the vapor phase ketonization of acetic acid with varying feed flow rates in the range of 0.1 to 0.3 mL min^–1^. As shown in Figure 13a, the percentage conversion of acetic acid on pristine zirconia increased with a corresponding increase in the residence time, from 55 to 95% on decreasing the feed flow rate from 0.3 to 0.1 mL min^–1^. It was observed that the selectivity to acetone decreased from 92 to 79% with the increase in acetic acid conversion, along with formation of diacetone alcohol and mesityl oxide as the byproducts. The byproducts in the process result from the self-condensation of acetone to diacetone alcohol and subsequent dehydration to mesityl oxide.^63^ The 0.1 SZ sulfated zirconia catalyst displayed relatively lower acetic acid conversion but improved selectivity to acetone (99%), as compared to the selectivity of 78% using pristine zirconia (Figure 13a). It was also observed that, on sulfated catalysts, high selectivity (∼99%) to acetone was achieved for all feed flow rates, unlike pristine zirconia, selectivity did not decrease upon increasing conversion and remained high. The significant reduction in the byproduct formation, from 22 to 1% at 0.1 mL min^–1^ feed flow rate of acetic acid, could be attributed to reduction of surface basicity, thus suppressing the self-condensation of acetone. Further, the selectivity of acetone for the pristine and sulfated surface was also compared at the similar conversion of acetic acid. As seen in Figure 13b, the selectivity to acetone using 1SZ catalyst was comparatively higher (95%) compared to the pristine zirconia (83%) at ∼80% conversion of acetic acid. The improved selectivity is attributed to diminished basic sites from the catalyst surface, thus inhibiting the side reaction of self-condensation of acetone. Similar observations are also reported by Sun et al. wherein surface basicity of ZnO modified ZrO_2_ leading to the side reaction.^64^ Sulfation reduces the concentration of basic sites, which is indicated both from experimental measurements using acid–base titration and CO_2_ TPD analysis as well as the theoretical studies (the section 3.3). On further increasing the concentration of sulfate species on the catalyst surface from 0.1SZ < 0.5SZ < 1SZ, acetic acid the percentage conversion increased linearly with increasing acidity, while the acetone selectivity remained high in the range of 96 to 99%. Figure 14 shows the normalized conversion of acetic acid as mmol converted per min per m^2^ of the sulfated catalysts at the feed flow rate of 0.1 mL min^–1^. The normalized conversion increased linearly with increasing the extent of sulfation on the catalyst surface. It was also observed from the CO_2_ TPD analysis of the catalysts, only the weak/moderate basic sites diminish upon sulfation of the catalysts, while strong basic sites are still present. It is plausible that the weak basic sites selectively facilitate more the self-condensation of acetone than the stronger basic sites, thus resulting in the improved selectivity to acetone upon sulfation.

In vapor phase ketonization, abstraction of a H_α_ is suggested as a plausible pathway for the acetic acid activation,^65^ which appears to be facilitated further with increasing surface acidity. The inhibition of byproducts formation, by aldol condensation of acetone is a direct consequence of the reduction of base strength. Thus, appropriate balance of acid–base sites resulting from sulfation of zirconia facilitates high selectivities in vapor phase ketonization of acetic acid to acetone.

## 4 Summary and Conclusions

We have presented a detailed study of the effect of sulfation on acid–base properties and activity/selectivity of zirconia catalysts in vapor phase ketonization of acetic acid. The sulfation of zirconia by using H_2_SO_4_ at different concentrations was shown to enhance the surface acidity and minimize the surface basicity of the catalysts. Pristine zirconia and sulfated zirconia prepared with H_2_SO_4_ solutions both showed tetragonal symmetry. Acetic acid ketonization in the vapor phase on pristine zirconia showed high conversions but lower selectivity to acetone due to subsequent condensation of acetone on basic sites. Sulfated zirconia catalysts showed significantly higher acetone selectivity (96–99%), as a result of inhibition of acetone self-condensation achieved by the reduction of number and strength of surface basic sites.

Dissociative adsorption of one and two H_2_SO_4_ molecules over t-ZrO_2_ (101) (approximately, 1 and 2 S/nm^2^) was investigated by DFT calculations for deeper insight into the most probable surface species in the sulfated catalyst. We have shown that tridentate SO_4_^2–^ presented the most stable structure within the analyzed systems. Furthermore, protons from the dissociative adsorption of H_2_SO_4_ are predicted to be on the surface of the catalyst, neighboring the SO_4_^2–^ group. When higher coverages were considered (∼2 SO_4_/nm^2^), both SO_4_^2–^ and HSO_4_^–^ species were obtained during structural optimization. Interestingly, the formation of HSO_4_^–^ had been considered unlikely in previous studies, due to the higher adsorption energies (i.e., more exothermic) observed for the fully deprotonated sulfates, as also observed in the present study. However, the formation of intermolecular interactions between neighboring adsorbed SO_4_^2–^ and HSO_4_^–^ species led to the stabilization of partially deprotonated adsorbates. This effect may be even more important when higher coverages (∼4 S/nm^2^) are considered, as the surface is unable to accommodate all adsorbed SO_4_^2–^ species in a tridentate fashion. Interestingly, such a surface configuration (comprising a mixture of SO_4_^2–^ and HSO_4_^–^ species) also agrees with the previously reported experimental characterization for these systems, with the presence of two distinct S-containing species, as reported by XANES, thermogravimetric analysis, and vibrational frequencies above 1400 cm^–1^, and this model could be an alternative to the hypothesis of pyrosulfate formation.

The possible formation of the dimeric species pyrosulfate under such conditions was also investigation by DFT. All the dimeric systems [S_2_O_7_^2–^,2 H^+^,H_2_O] obtained in this investigation had a higher energy than those of the isolated SO_4_^2–^ species (monomeric system). When compared to the reference system (**4(r)**), the relative energy of [S_2_O_7_^2–^,2 H^+^,H_2_O] varied between 20.0 and 44.0 kcal mol^–1^. Additionally, the calculated energy barriers for the dimerization of SO_4_^2–^ species ranged between 60.0 and 70.0 kcal mol^–1^, when compared to the same reference systems. These results indicate that formation of dimeric species would be unlikely to occur in such low coverage systems, even under calcination conditions. Furthermore, under mild condition and in a water-rich environment, the hydrolysis of the dimeric species (if present, at all) would be favored to produce isolated SO_4_^2–^ on the surface of the catalyst.

The changes in acidity upon sulfation were investigation by titration of pristine and sulfated zirconia, which has clearly shown an increase of acidity when higher concentrations of H_2_SO_4_ solution were used in the synthesis of the catalyst. Computed proton transfer energy and adsorption of base probe molecules (pyridine and NH_3_) on the model surface showed similar results to those observed by titration, with significant increase of Brønsted and Lewis acidity of this catalyst. In addition, the basicity of the surface was significantly reduced upon sulfation as shown by CO_2_ TPD analysis and the computed adsorption energies for CO_2_ capture by the clean and sulfated surfaces. Finally, the effect of sulfation over the activity and selectivity of zirconia in the ketonization of acetic acid was investigated for the synthesized catalysts. Although conversion rates for such reactions were observed to drop significantly for the sulfated catalysts, their selectively to form ketones was observed to be improved.

Overall, these results provide a thorough description of sulfated zirconia structure, the identity of the most likely surface species under hydrating and mild conditions, its acid–base properties, and activity and selectivity of sulfated zirconia in ketonization reactions. The insight presented here will be key in the identification of new modifications of zirconia for optimization of acidity and basicity properties for new applications in catalysis.

## Supporting Information Available

1. Construction of t-ZrO_2_ (101) slab model; 2. alternative structure configurations for H_2_SO_4_/t-ZrO_2_(101) system; 3. alternative structure configurations for adsorption of a 2nd H_2_SO_4_ over H_2_SO_4_/t-ZrO_2_(101) system; 4. alternative structure configurations for [2H^+^,S_2_O7_2_^–^] over t-ZrO_2_ (101); 5. vibrational frequency calculations (PDF)
