# Supplementary material for: Role of Sulfation of Zirconia Catalysts in Vapor Phase Ketonization of Acetic Acid
Source: J Phys Chem C Nanomater Interfaces. 2021 Dec 13;125(50):27578–95. doi: 10.1021/acs.jpcc.1c06920 (PMC8713292; doi:10.1021/acs.jpcc.1c06920)
Supplement: Supplementary file 1 — jp1c06920_si_001.pdf [file jp1c06920_si_001.pdf]

On the Role of Sulfation of Zirconia Catalysts in Vapour Phase Ketonization of Acetic Acid

Maicon Delarmelina*[a,b], Gunjan Deshmukh[b,c], Alexandre Goguet[b,c], C. Richard A. Catlow[a,b, d], Haresh Manyar*[b,c]

[a]School of Chemistry, Cardiff University, Main Building, Park Place, Cardiff, CF10 3AT, UK.

[b]UK Catalysis Hub, Research Complex at Harwell, STFC Rutherford Appleton Laboratory, Didcot, Oxfordshire, OX11 0FA, UK.

[c]School of Chemistry and Chemical Engineering, Queen's University Belfast, David-Keir Building, Stranmillis Road, Belfast, BT9 5AG, UK.

[d]Department of Chemistry, University College London, 20 Gordon St., London WC1 HOAJ, UK.

E-mail: DelarmelinaM@cardiff.ac.uk, H.Manyar@qub.ac.uk

Table of Contents

1. Construction of t-ZrO_2 (101) slab model S2

2. Alternative structure configurations for H_2SO_4/t-ZrO_2(101) system S3

3. Alternative structure configurations for adsorption of a 2nd H_2SO_4 over H_2SO_4/t-ZrO_2(101) system........ S7

4. Alternative structure configurations for [2 H^+,$S_2O_7^{2-}$] over t-ZrO_2 (101) S11

5. Vibrational Frequency Calculations S12

1. Construction of t-ZrO_2 (101) slab model

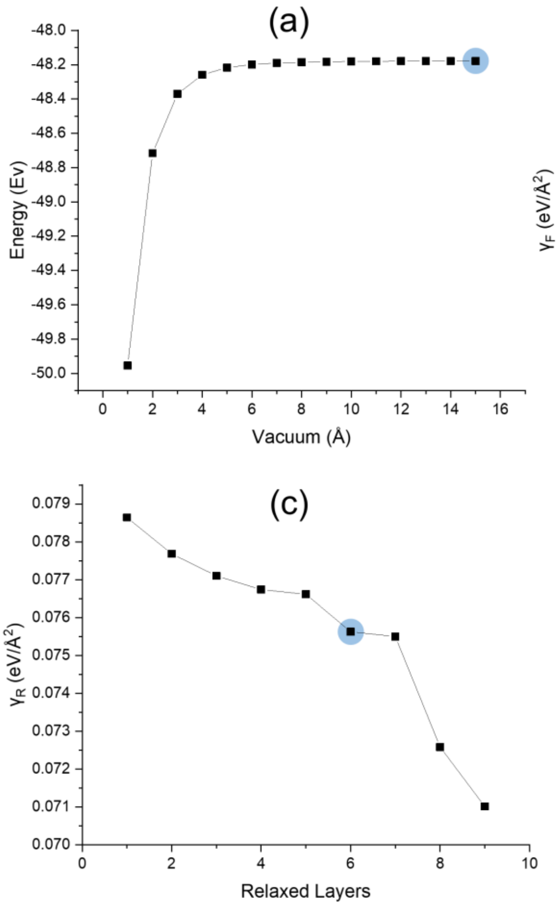

Figure S1. Vacuum size (a), slab thickness (b), and relaxed atomic layers (c) tested for the construction of t-ZrO_2 (101) surface slab model. Highlighted graph points indicate selected choices.

2. Alternative structure configurations for H_2SO_4/t-ZrO_2(101) system

Table S1. Alternative structures for dissociate adsorption of a H_2SO_4 at adsorption sites “A” and “B” of structure **3(g)**.

Entry	Initial structure	After optimization	Obs.	$E_{relative}$ (kcal mol^{-1})
**3(a)**	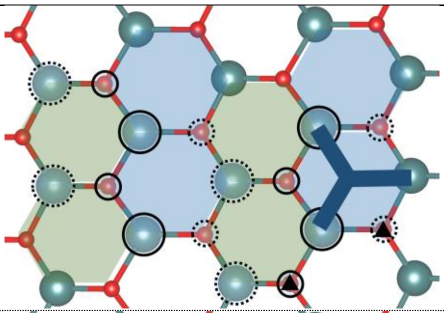	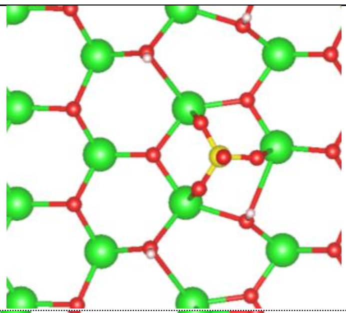	-	8.11
**3(b)**	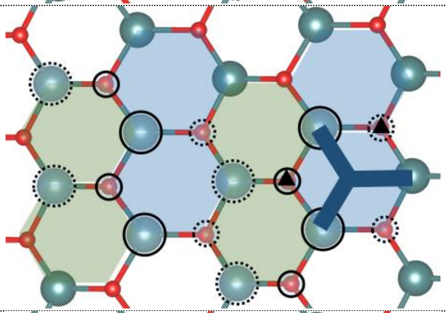	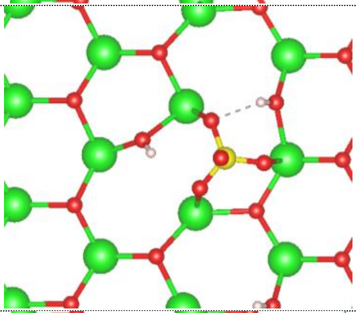	-	2.79
**3(c)**	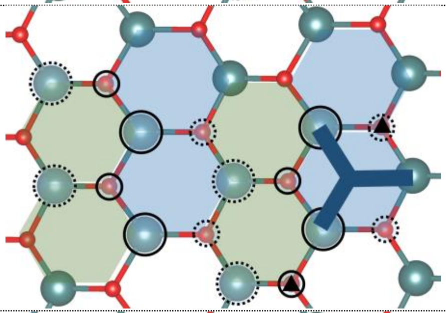	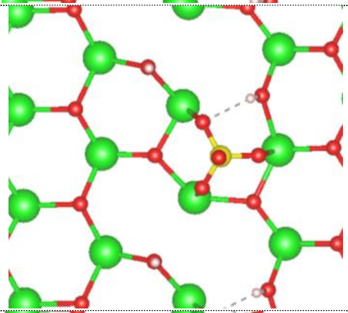		6.89
**3(d)**	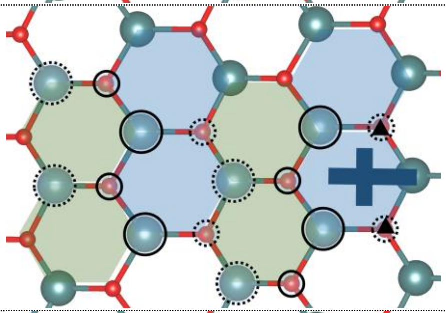	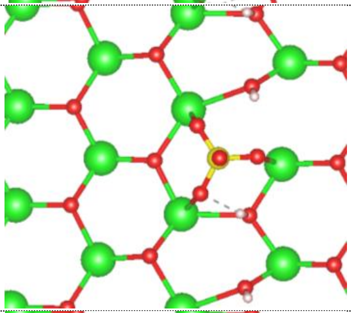	Change from bi- to tridentate SO4	27.16
**3(e)**	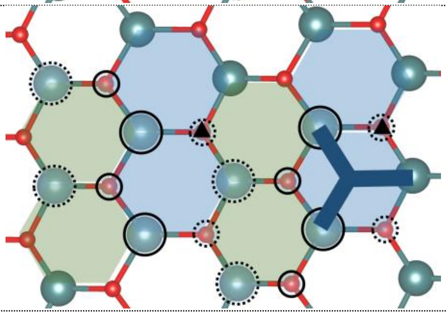	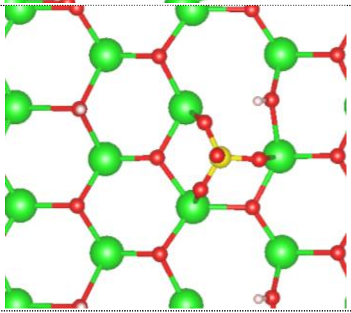	-	19.30

**3(f)**	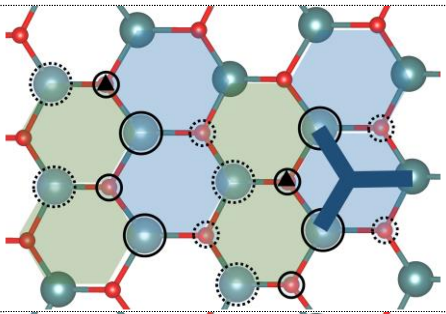	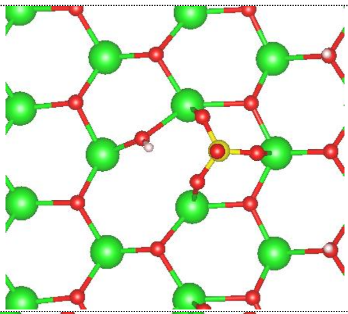	-	2.96
**3(g)**	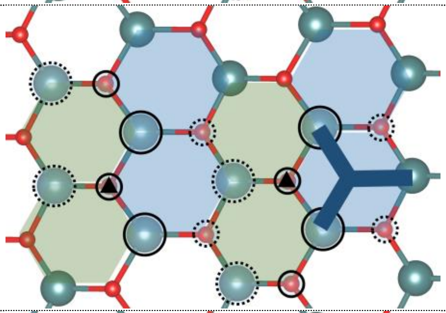	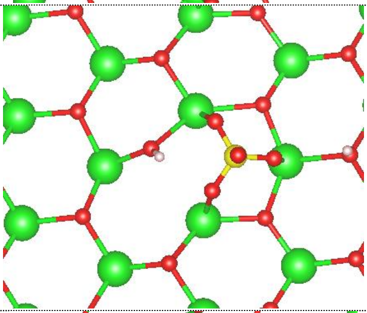	-	0.00
**3(h)**	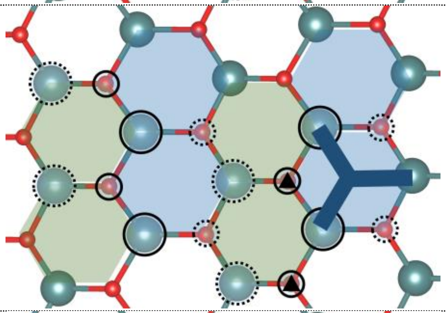	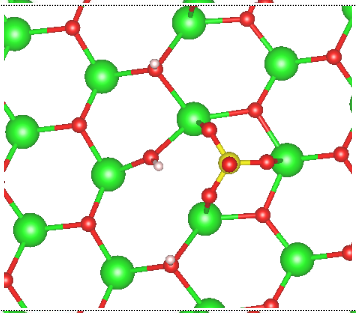	-	3.45
**3(i)**	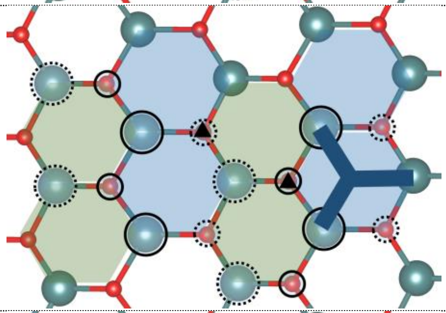	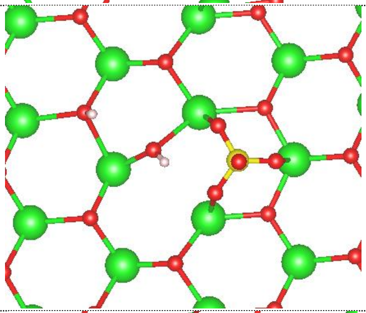	-	12.17
**3(j)**	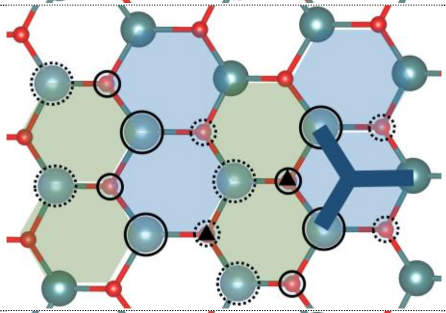	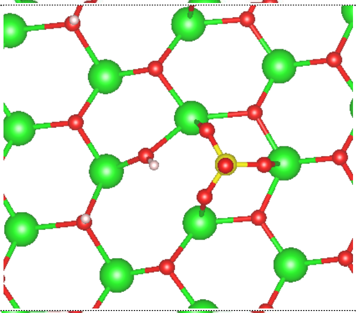	-	12.32
**3(k)**	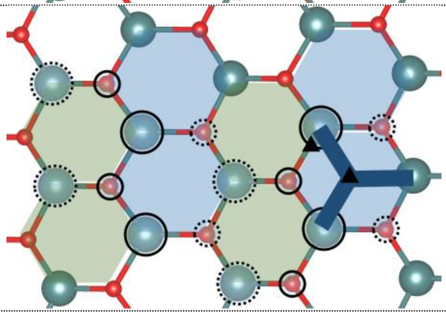	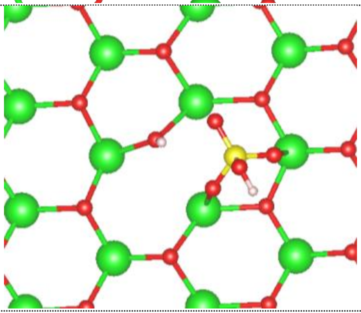	-	25.61

**3(l)**	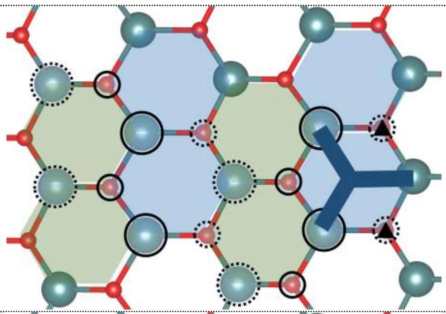	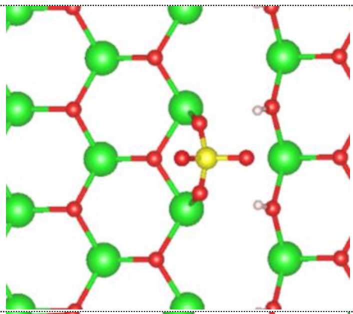	-	16.33
**3(m)**	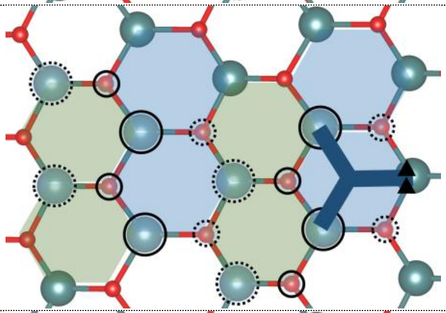	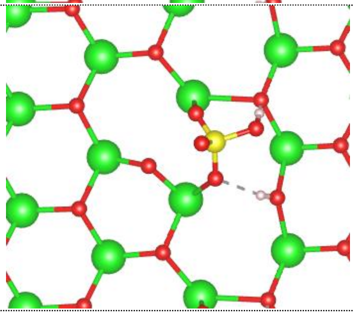	H abstraction; S-OH spin; Bidentate SO4 is obtained with SOH···Zr interaction being formed	27.49
**3(n)**	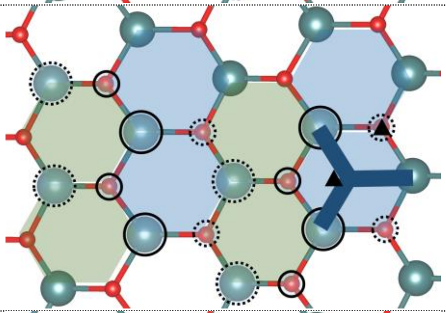	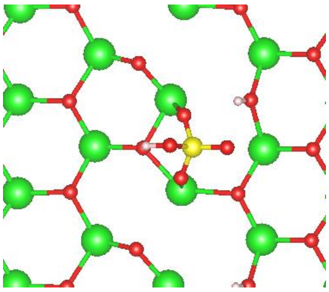	-	30.95
**3(r)**	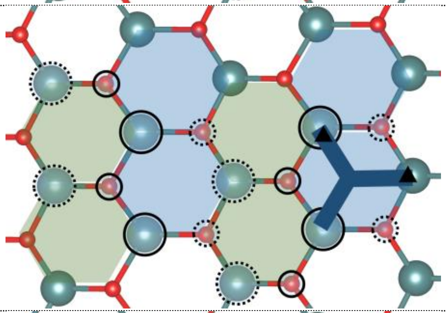	-	Desorption	-
**3(s)**	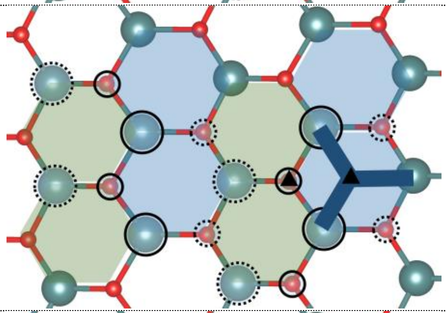	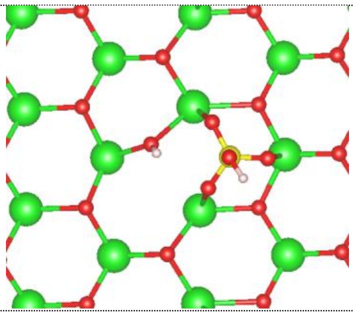	Equivalent to **3(k)**	24.61
**3(t)**	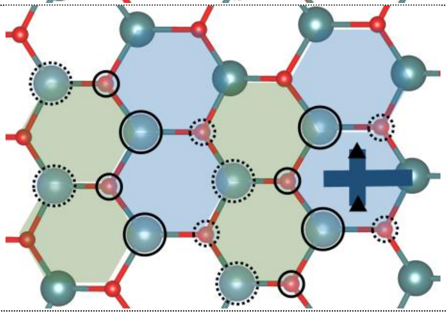	-	Desorption	-

**3(u)**	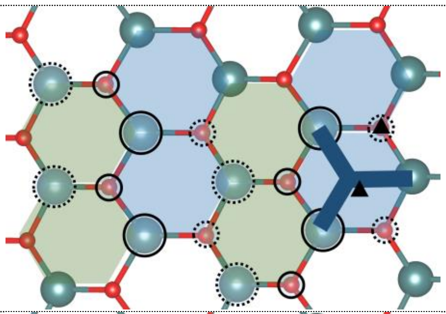	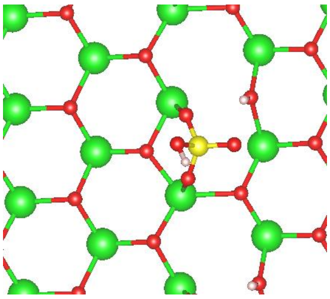	Equivalent to **3(n)**	31.07
**3(v)**	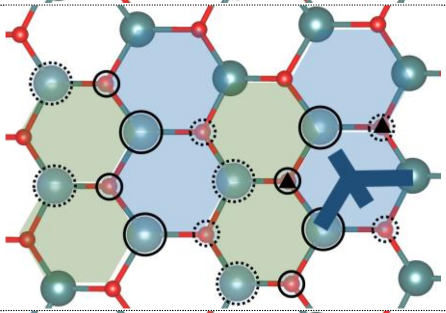	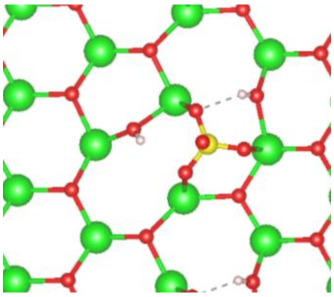	Equivalent to **3(b)**	-
**3(w)**	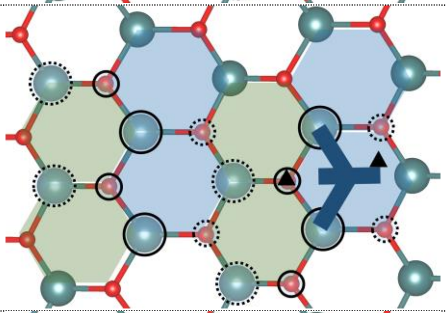	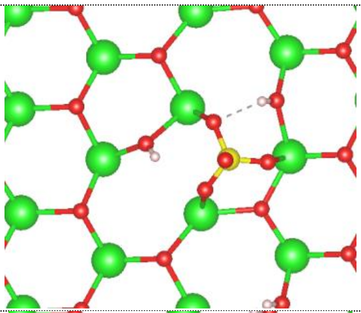	Equivalent to **3(b)**	-
**3(x)**	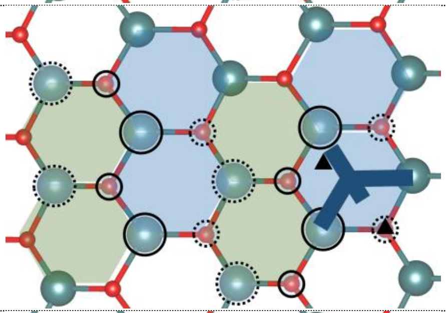	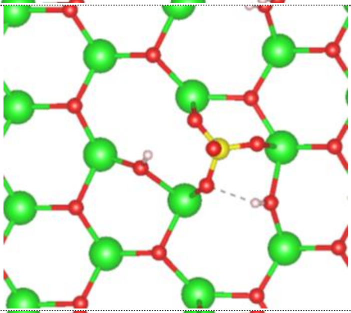	Equivalent to **3(b)**	-
**3(y)**	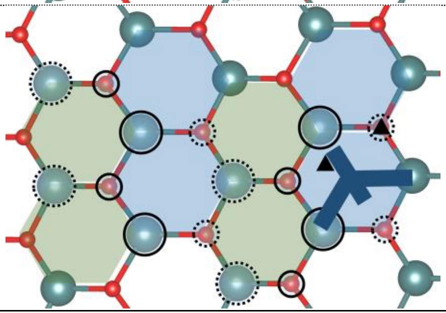	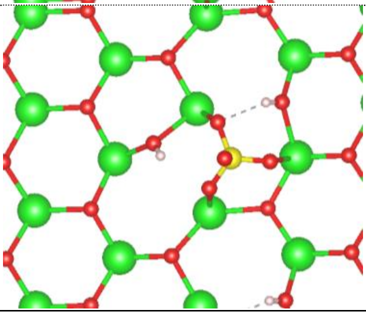	Equivalent to **3(b)**	-

3. Alternative structure configurations for adsorption of a 2nd H_2SO_4 over H_2SO_4/t-ZrO_2(101) system

Table S2. Alternative structures for dissociate adsorption of a 2nd H_2SO_4 at site "B" of structure **3(g)**.

Entry	Initial structure	After optimization	$E_{relative}$ (kcal mol^{-1})
**5(a)**	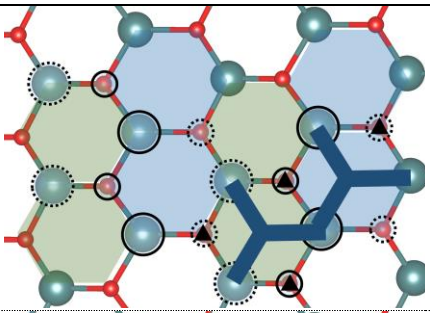	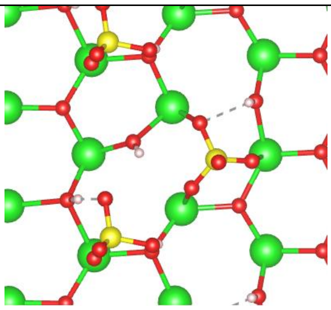	8.58
**5(b)**	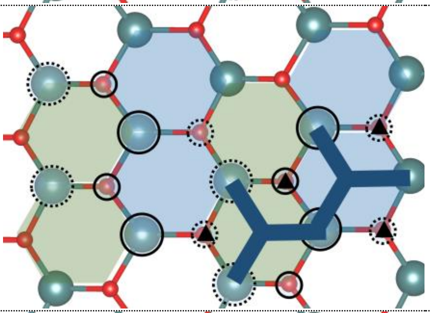	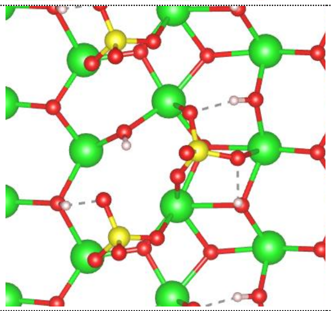	39.72
**5(c)**	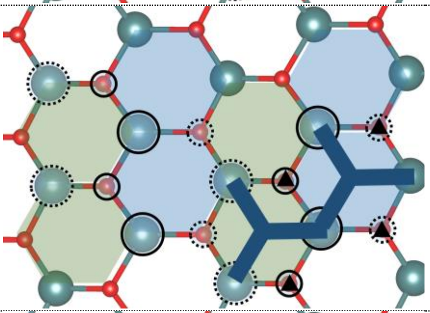	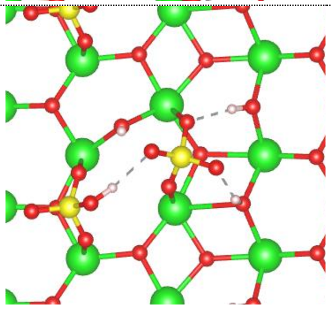	6.34
**5(d)**	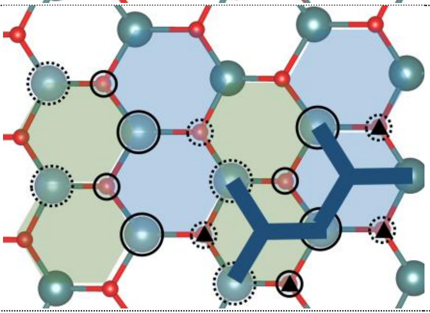	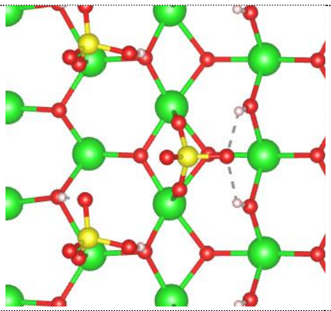	19.17

**5(e)**	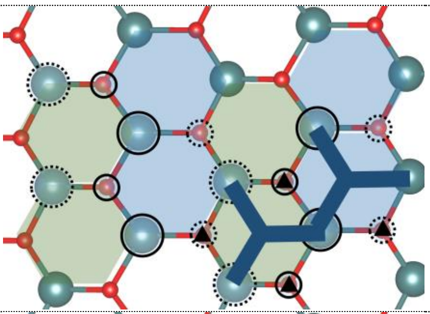	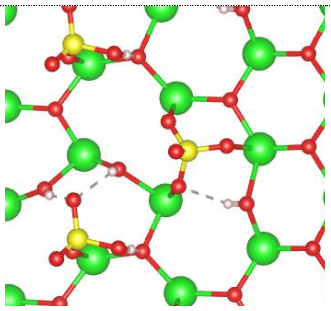	4.29
**5(f)**	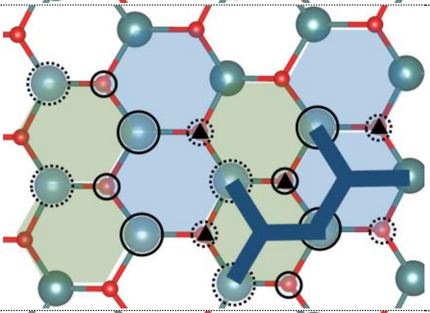	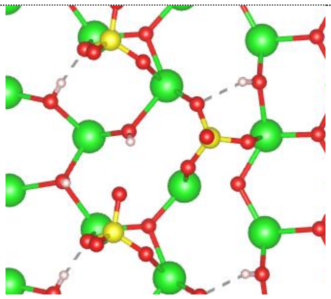	24.05
**5(g)**	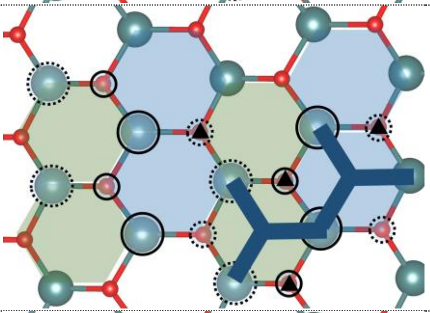	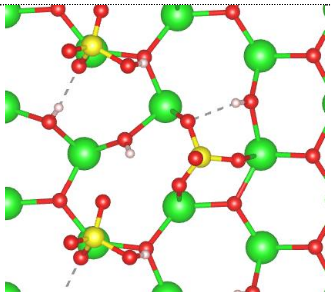	0.62
**5(h)**	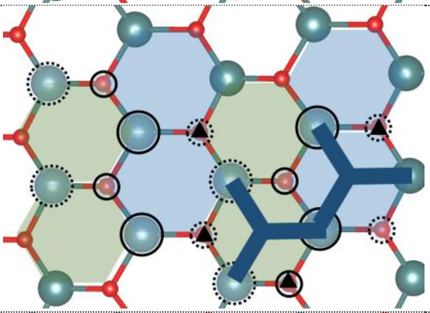	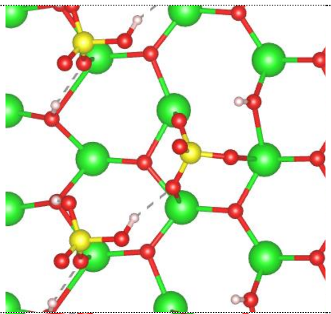	12.87
**5(i)**	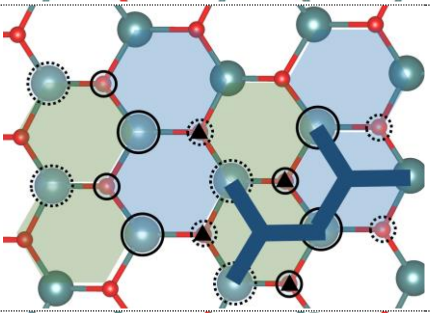	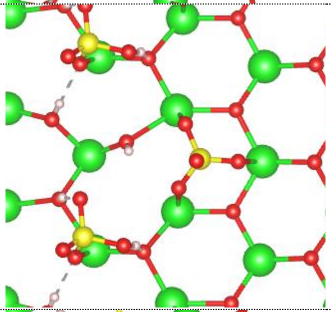	10.01
**5(j)**	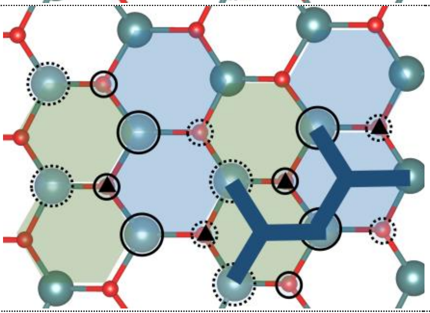	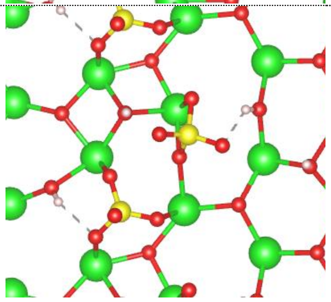	16.22

**5(k)**	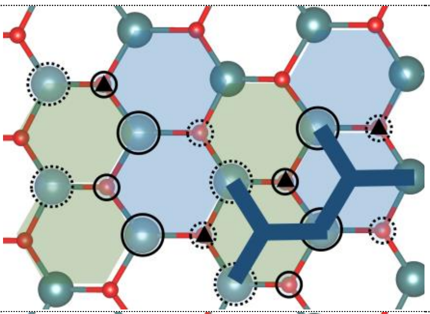	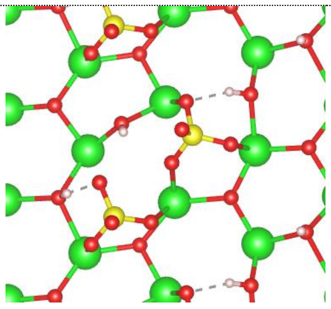	33.03
**5(l)**	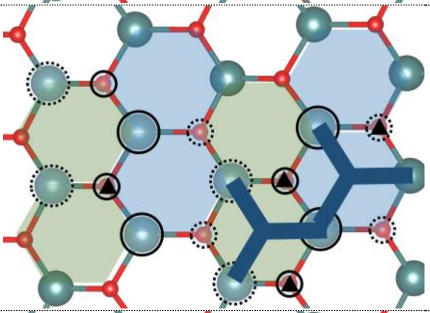	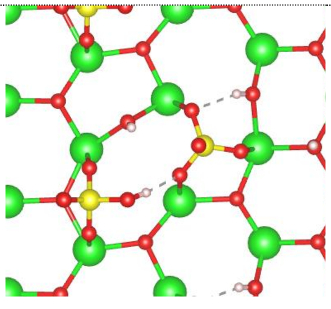	0.00
**5(m)**	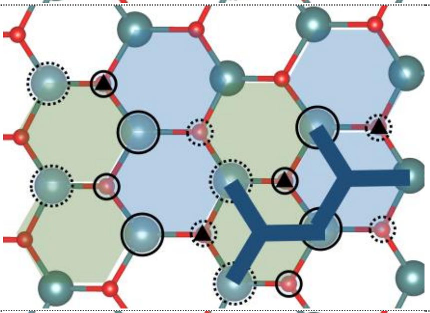	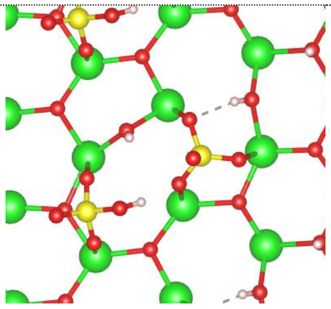	33.02
**5(n)**	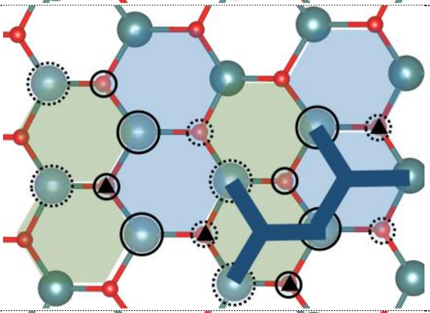	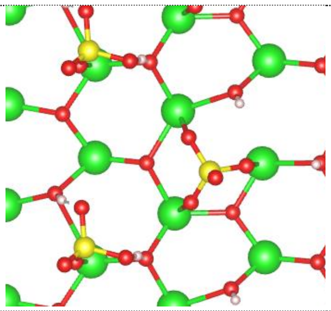	11.80
**5(o)**	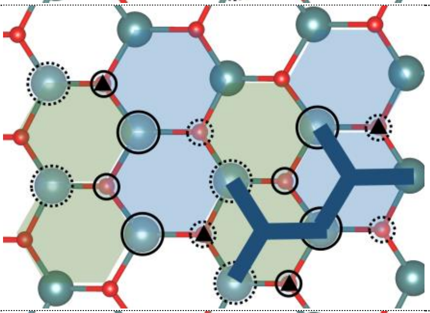	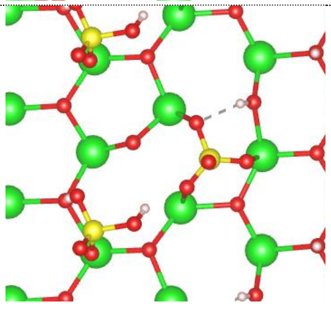	20.74
**5(p)**	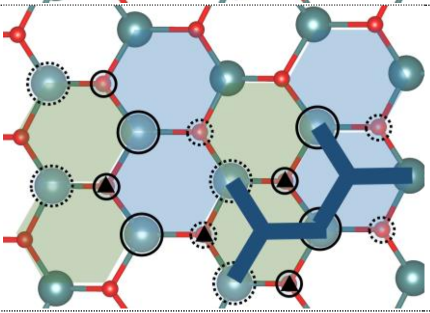	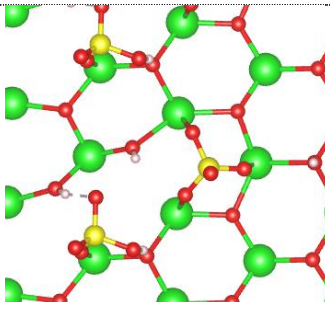	1.14

**5(q)**	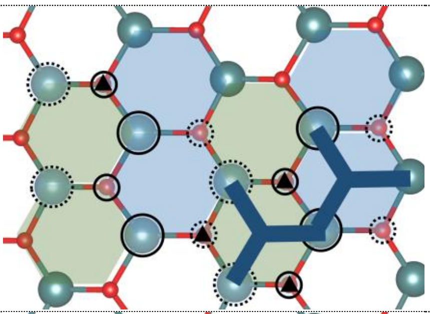	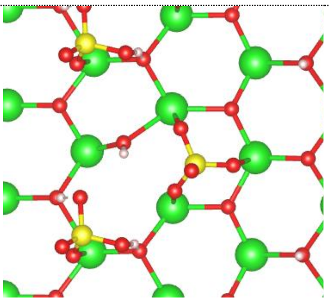	5.19
**5(r)**	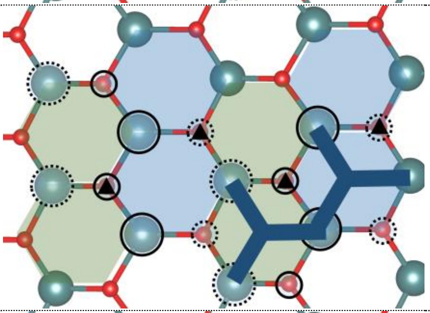	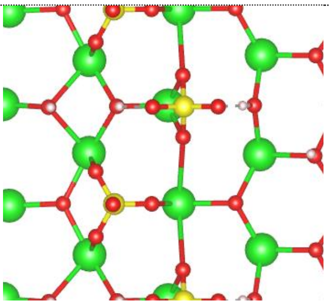	16.29
**5(s)**	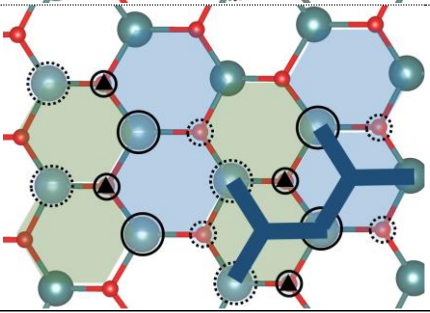	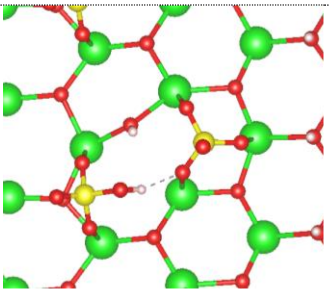	4.00

4. Alternative structure configurations for [2 H^+,$S_2O_7^{2-}$] over t-ZrO_2 (101)

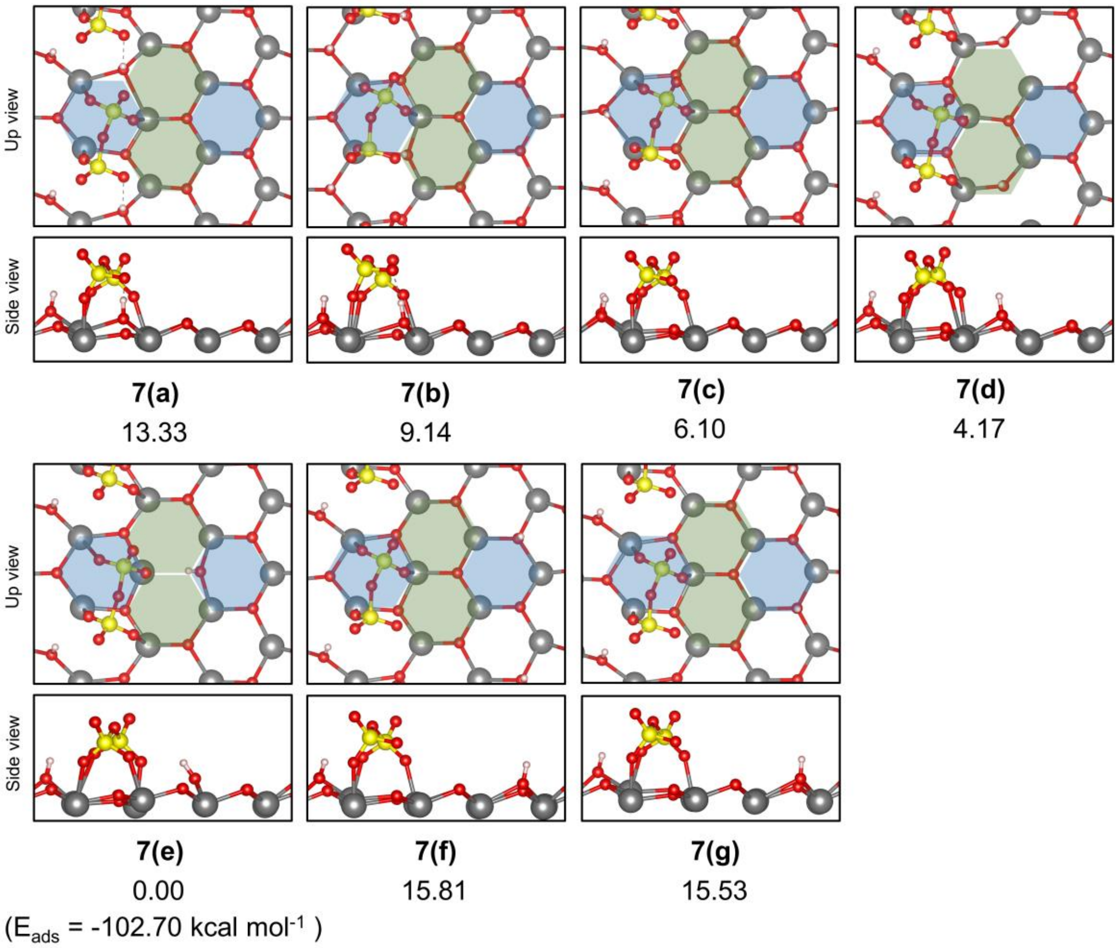

Figure S2. Relative energy of the most stable structures identified for dissociative adsorption of $H_2S_2O_7$ over t-ZrO_2 (101). Arrangement of $S_2O_7^{2-}$ anion was considered based on structure **6(f)**. Total energy of structure **7(e)** was taken as reference to calculate the given relative energies.

5. Vibrational Frequency Calculations

Vibrational frequencies were calculated for selected CO_2- and pyridine-containing surfaces (**Tables S3-S6**). The most stable structures were selected accordingly to the results presented in the main text (**Figure 11** and **Tables 4** and **6**). Vibrational frequency calculations were performed at the gamma-point for the fully optimized structures by using the standard finite differences approach. Infrared intensities were obtained from the Born effective charges using VASP-infrared-intensity bash script implemented by D. Karhanek (https://github.com/dakarhanek/VASP-infrared-intensities, Access: October/2021).

Table S3. Calculated frequencies ($\tilde{\nu}$, cm^{-1}) and infrared intensities for most stable adsorption modes of pyridine over clean t-ZrO_2 (111) surface, according to results presented in **Figure 11**.

**(a), Figure 11**		**(b), Figure 11**		**(c), Figure 11**		**(d), Figure 11**	
ν	IR intens.	ν	IR intens.	ν	IR intens.	ν	IR intens.
3144	0.070	3139	0.121	3137	0.072	3134	0.206
3135	0.024	3131	0.184	3130	0.048	3128	0.109
3127	0.044	3120	0.352	3117	0.106	3120	0.114
3120	0.184	3113	0.287	3107	0.074	3106	0.061
3108	0.056	3107	0.126	3092	0.072	3096	0.627
1587	0.256	1586	0.457	1582	0.235	1583	0.417
1554	0.062	1553	0.072	1555	0.061	1553	0.063
1472	0.214	1470	0.438	1469	0.212	1472	0.591
1428	0.198	1427	0.840	1426	0.288	1427	0.837
1349	0.038	1346	0.089	1345	0.056	1348	0.046
1276	0.016	1277	0.027	1276	0.009	1275	0.031
1219	0.137	1216	0.285	1214	0.134	1217	0.229
1151	0.019	1152	0.032	1151	0.016	1155	0.027
1066	0.033	1068	0.093	1065	0.103	1065	0.009
1061	0.213	1062	0.410	1059	0.160	1060	0.549
1021	0.116	1024	0.178	1019	0.116	1020	0.237
1001	0.020	1003	0.112	998	0.053	998	0.009
1000	0.074	1001	0.091	996	0.040	996	0.012
988	0.004	993	0.008	996	0.016	994	0.151
955	0.030	964	0.033	964	0.028	965	0.037
868	0.000	873	0.001	875	0.001	877	0.002
748	0.487	747	0.481	749	0.656	750	0.532
697	1.000	701	1.000	705	1.000	704	1.000
645	0.215	659	0.996	647	0.072	652	0.056

Table S4. Calculated frequencies ($\tilde{\nu}$, cm^{-1}) and infrared intensities for most stable adsorption modes of pyridine over sulfated t-ZrO_2 (111) surface, according to results presented in **Table 4**.

**(Zr1-O3), Table 4**		**(Zr2-O4), Table 4**		**(Zr3-O4), Table 4**		**(Zr4-O6), Table 4**		**(Zr8-O2), Table 4**	
$\tilde{\nu}$	IR intens.	$\tilde{\nu}$	IR intens.	$\tilde{\nu}$	IR intens.	$\tilde{\nu}$	IR intens.	$\tilde{\nu}$	IR intens.
3707	0.507	3760	0.562	3764	0.412	3762	0.466	3761	0.873
3496	1.000	3648	1.000	3612	1.000	3651	1.000	3673	1.000
3170	0.080	3157	0.011	3143	0.008	3155	0.022	3157	0.011
3140	0.002	3141	0.013	3133	0.010	3138	0.003	3152	0.047
3135	0.012	3136	0.007	3126	0.006	3133	0.009	3140	0.001
3126	0.028	3127	0.025	3118	0.001	3112	0.003	3131	0.007
3112	0.007	3112	0.010	3108	0.004	3094	0.014	3115	0.009
1590	0.057	1588	0.106	1576	0.057	1586	0.082	1588	0.109
1553	0.010	1553	0.012	1554	0.008	1554	0.015	1558	0.024
1477	0.066	1473	0.075	1464	0.059	1474	0.073	1471	0.106
1432	0.054	1429	0.108	1424	0.124	1429	0.086	1429	0.124
1358	0.007	1350	0.013	1338	0.000	1352	0.006	1344	0.020
1274	0.002	1278	0.003	1278	0.005	1276	0.002	1280	0.001
1244	0.463	1237	0.826	1247	0.704	1241	0.773	1239	0.895
1226	0.007	1219	0.015	1209	0.016	1219	0.002	1216	0.020
1158	0.004	1155	0.005	1149	0.004	1155	0.003	1156	0.007
1070	0.005	1070	0.007	1064	0.008	1069	0.003	1071	0.076
1064	0.101	1060	0.109	1060	0.048	1060	0.087	1063	0.104
1055	0.279	1024	0.027	1031	0.504	1030	0.707	1048	0.829
1024	0.020	1018	0.947	1023	0.011	1023	0.045	1029	0.052
1004	0.172	1003	0.040	992	0.018	1000	0.035	1000	0.154
1003	0.366	1000	0.002	991	0.447	997	0.000	993	0.750

Table S5. Calculated frequencies ($\tilde{\nu}$, cm^{-1}) and infrared intensities for most stable adsorption modes of CO_2 over clean t-ZrO_2 (111) surface, according to results presented in **Figure 11**.

**(i), Figure 11**		**(j), Figure 11**		**(k), Figure 11**		**(l), Figure 11**		**(m), Figure 11**	
$\tilde{\nu}$	IR intens.	$\tilde{\nu}$	IR intens.	$\tilde{\nu}$	IR intens.	$\tilde{\nu}$	IR intens.	$\tilde{\nu}$	IR intens.
1746	1.000	1730	1.000	1728	1.000	1391	0.930	1392	0.962
1146	0.748	1137	0.959	1121	0.901	1381	1.000	1378	1.000
848	0.478	906	0.456	895	0.453	1072	0.015	1070	0.017
778	0.057	783	0.050	782	0.053	829	0.030	830	0.033
688	0.039	698	0.038	695	0.036	650	0.003	651	0.006
604	0.001	623	0.182	620	0.024	648	0.004	650	0.007
600	0.019	618	0.120	614	0.051	614	0.016	637	0.000
595	0.037	610	0.003	612	0.101	607	0.003	620	0.091
587	0.237	608	0.000	603	0.108	605	0.003	619	0.043
577	0.011	582	0.072	586	0.135	596	0.037	600	0.037
569	0.001	577	0.004	578	0.024	572	0.010	589	0.009
564	0.004	570	0.068	572	0.019	571	0.011	581	0.077
561	0.090	564	0.009	568	0.095	568	0.135	580	0.440
550	0.083	554	0.332	561	0.181	562	0.049	573	0.007
539	0.043	549	0.123	558	0.542	535	0.133	555	0.125
519	0.331	541	0.570	540	0.046	533	0.193	553	0.083
516	0.324	536	0.216	537	0.242	527	0.006	539	0.023
511	0.203	534	0.011	533	0.086	514	0.081	531	0.142
498	0.021	520	0.213	514	0.256	513	0.062	523	0.061
492	0.144	509	0.019	508	0.070	502	0.021	523	0.079
484	0.073	500	0.009	492	0.018	490	0.010	505	0.044
474	0.101	490	0.003	491	0.026	490	0.061	503	0.000
471	0.061	486	0.299	475	0.020	485	0.144	482	0.018
459	0.044	472	0.016	464	0.141	470	0.133	460	0.102
451	0.002	458	0.084	456	0.073	468	0.235	453	0.209
445	0.099	441	0.212	434	0.057	464	0.012	443	0.083

Table S6. Calculated frequencies ($\tilde{\nu}$, cm^{-1}) and infrared intensities for most stable adsorption modes of CO_2 over sulfated t-ZrO_2 (111) surface, according to results presented in **Table 6**.

η^2-CO_2								η^3-CO_2							
**O1-Zr2, Table 6**		**O1-Zr1, Table 6**		**O2-Zr1, Table 6**		**O3-Zr4, Table 6**		**O1, Table 6**		**O2, Table 6**		**O3, Table 6**		**O4, Table 6**	
$\tilde{\nu}$	IR intens.	$\tilde{\nu}$	IR intens.	$\tilde{\nu}$	IR intens.	$\tilde{\nu}$	IR intens.	$\tilde{\nu}$	IR intens.	$\tilde{\nu}$	IR intens.	$\tilde{\nu}$	IR intens.	$\tilde{\nu}$	IR intens.
3772	0.273	3778	0.246	3773	0.266	3776	0.220	3773	0.201	3789	0.157	3765	0.151	3774	0.126
3703	0.261	3700	0.297	3699	0.257	3691	0.433	3691	0.184	3659	0.101	3726	0.094	3709	0.163
1744	0.926	1760	0.757	1750	0.854	1765	0.931	1514	1.000	1393	0.925	1406	1.000	1416	1.000
1272	0.531	1273	0.437	1275	0.515	1270	0.522	1293	0.778	1385	1.000	1381	0.961	1379	0.940
1127	0.813	1137	0.688	1128	0.764	1162	0.797	1265	0.369	1264	0.329	1265	0.309	1264	0.325
968	0.518	1011	0.392	1007	0.376	1015	0.342	1044	0.083	1070	0.012	1075	0.012	1077	0.010
915	0.377	935	0.083	953	0.240	966	0.297	974	0.419	978	0.307	1001	0.207	963	0.266
897	0.382	876	0.151	883	0.634	892	1.000	922	0.300	916	0.162	931	0.077	926	0.358
865	0.153	872	1.000	873	1.000	880	0.190	896	0.164	897	0.432	891	0.423	897	0.112
857	0.857	848	0.454	869	0.041	855	0.459	864	0.439	879	0.326	861	0.389	868	0.356
844	1.000	844	0.495	841	0.483	845	0.704	847	0.737	830	0.023	829	0.042	830	0.596
780	0.128	775	0.093	778	0.094	771	0.111	819	0.075	820	0.280	821	0.590	828	0.034
695	0.028	698	0.015	700	0.027	702	0.024	702	0.013	702	0.239	695	0.045	671	0.100
652	0.256	648	0.169	665	0.134	650	0.081	658	0.055	675	0.042	670	0.111	662	0.006
625	0.031	637	0.364	644	0.072	632	0.031	645	0.109	665	0.084	658	0.107	656	0.079
621	0.115	631	0.076	635	0.253	626	0.102	636	0.045	658	0.013	653	0.006	653	0.023
611	0.136	617	0.004	631	0.005	618	0.035	630	0.120	649	0.005	643	0.047	644	0.002
609	0.075	616	0.086	619	0.100	615	0.023	622	0.064	641	0.011	636	0.026	628	0.166
606	0.051	610	0.058	617	0.103	607	0.037	621	0.081	640	0.016	622	0.021	627	0.019
600	0.114	601	0.004	610	0.010	603	0.065	612	0.124	622	0.078	617	0.030	615	0.156
598	0.068	598	0.018	608	0.004	602	0.112	608	0.032	618	0.060	614	0.095	608	0.052
589	0.019	586	0.014	591	0.002	594	0.081	598	0.006	607	0.024	607	0.007	596	0.017
584	0.252	584	0.061	588	0.226	590	0.030	592	0.181	597	0.327	597	0.042	593	0.019
578	0.091	582	0.038	585	0.166	579	0.038	584	0.022	590	0.008	589	0.098	586	0.042
